# Variety-Specific Responses of Mulberry to Copper-Induced Stress

**DOI:** 10.3390/plants15193070

**Published:** 2026-10-08

**Authors:** Jan Senekovič, Anna Gasperl, Mario Lešnik, Andreja Urbanek Krajnc

**Affiliations:** 1 Faculty of Agriculture and Life Sciences, University of Maribor, Pivola 10, 2311 Hoče, Slovenia; jan.senekovic1@um.si (J.S.); mario.lesnik@um.si (M.L.); 2 Department of Plant Sciences, Stress- and Cell Biology of Plants, Institute of Biology, University of Graz, 8010 Graz, Austria; anna.gasperl@uni-graz.at

**Keywords:** copper, glutathione–ascorbate cycle, heavy-metal stress, mulberry, oxidative stress, phenolic compounds, photosynthetic pigments, stress tolerance, xanthophyll cycle

## Abstract

Copper (Cu) is an essential plant micronutrient, but in excessive amounts it becomes phytotoxic, making Cu contamination of agricultural soils an important environmental concern. Because Cu tolerance mechanisms are complex and often genotype-specific, there is growing interest in multipurpose crops capable of thriving under metal stress. Mulberry is one of such species, which enables the combination of diverse agricultural uses with notable tolerance to heavy metals. In the present study, the tolerance to Cu-induced stress of five varieties belonging to *Morus alba* and *M. indica* was assessed in a pot experiment, in which the trees were watered with a CuSO_4_ solution that increased the soil Cu concentration to 150 mg kg^−1^, while the plants in the control group were watered with the same volume of tap water. In leaves sampled on days 0, 3, 7, and 21 after CuSO_4_ treatment, Cu content was analyzed by AAS; ascorbate, dehydroascorbate, thiols, oxidized thiols and xanthophyll pigments were analyzed by HPLC; and total phenolic content and photosynthetic pigment content were determined spectrophotometrically. The effect of CuSO_4_ treatment on the maximum quantum yield of photosystem II was analyzed by measuring the chlorophyll fluorescence of leaves on day 21 after CuSO_4_ treatment. The results showed a species-specific response to Cu-induced stress. Leaves of *M. alba* varieties exhibited a more pronounced increase in Cu content, higher antioxidant levels, more efficient redox regulation, a smaller decline in photosynthetic pigment contents and photosynthetic efficiency, resulting in more effective photoprotection through the xanthophyll cycle when compared to *M. indica* varieties under the conditions of this experiment. In particular, the varieties ‘Florio’ and ‘Giazzola’ exhibited high Cu tolerance and therefore show potential for cultivation in Cu-contaminated soils.

## 1. Introduction

Copper (Cu) is an essential micronutrient and an environmental pollutant [1]. As a cofactor, it is involved in several plant physiological processes, including photosynthesis, mitochondrial respiration, carbohydrate metabolism, formation of phenolics, superoxide scavenging, cell wall remodelling, and ethylene perception [2]. Cu is widely used in agriculture as a fertilizer and for plant disease management, while organic agriculture particularly relies on Cu-containing fungicides [3]. Repeated application of Cu-based products has contributed to elevated Cu levels in agricultural soils [4,5]. Copper sulfate (CuSO_4_), including its use in Bordeaux mixture, represents an important source of Cu-contamination [6,7,8]. Excess Cu can contribute to soil degradation and phytotoxicity, negatively affect soil microbial functions, fertility and biodiversity, and, through leaching, affect surface and ground waters [9]. High Cu concentrations can also reduce the suitability of contaminated soils for agricultural production [10].

In plants, excessive Cu induces oxidative damage through enhanced production of reactive oxygen species (ROS), affecting growth, photosynthesis, membrane integrity, thylakoid structure, and photosystem II (PSII) function [11,12,13]. Typical symptoms include stunted growth, chlorosis, leaf deformation and necrosis [14]. Cu concentrations above 100 mg kg^−1^ soil can cause toxic effects in contaminated soils, although the toxicity threshold varies considerably among plant species [15]. Cu toxicity also differs among genotypes within the same species, resulting in substantial variation in tolerance [16]. Because leaves reflect physiological and metabolic responses related to whole-plant tolerance, their analysis can provide valuable information on plant responses to Cu stress [16,17].

Plants possess coordinated enzymatic and non-enzymatic antioxidant defence systems that control oxidative processes and protect cells from damage [18]. Non-enzymatic antioxidants, including ascorbate (ASC), glutathione (GSH), carotenoids, and phenolic compounds, contribute to plant tolerance to abiotic stress [19]. The ascorbate–glutathione (ASC–GSH) cycle is a key component of redox regulation under Cu-induced stress [20]. ASC contributes to ROS detoxification and serves as a substrate for ascorbate peroxidase (APX) [21,22]. GSH participates in hydrogen peroxide detoxification and regeneration of reduced ASC through dehydroascorbate reductase (DHAR) and also acts as a reducing agent and substrate for glutathione peroxidase (GPX) and glutathione-S-transferases (GST) [23]. In addition, GSH is a precursor of phytochelatins, which participate in the detoxification of heavy metals such as Cu, cadmium (Cd), and zinc (Zn) through metal chelation and subsequent vacuolar accumulation [24]. Changes in the size and redox status of ASC and GSH pools may therefore indicate the capacity of different genotypes to maintain redox homeostasis under Cu stress.

Phenolic compounds represent another component of the antioxidant response. The phenylpropanoid pathway and its derived compounds contribute to stress tolerance and increased phenolic accumulation is frequently observed under heavy-metal and Cu-induced stress [25,26]. Phenolics can scavenge ROS and interact directly with metal ions, reducing metal-catalyzed oxidative reactions [27]. Flavonoids can additionally chelate Cu ions and may contribute to the modulation of Cu-induced oxidative stress [28]. The coordinated response of ASC, GSH, thiols and phenolic compounds may therefore contribute to maintaining cellular redox balance under excessive Cu exposure.

Cu-induced oxidative stress is also closely associated with impairment of the photosynthetic apparatus. Excess Cu can reduce photosynthetic pigment contents and inhibit photosynthesis and the maximum quantum yield of PSII (Fv/Fm) [29,30]. These effects have been associated with impaired chlorophyll (chl) biosynthesis, chloroplast damage, and disruption of photosynthetic electron transport, particularly at PSII, further contributing to ROS formation and oxidative damage [16]. Carotenoids provide photoprotection under heavy-metal stress by protecting chl and participating in the dissipation of excess excitation energy [31]. The xanthophyll cycle contributes to non-photochemical energy dissipation and can be activated under Cu stress [32]. Excess Cu has been reported to stimulate violaxanthin de-epoxidation and zeaxanthin accumulation, indicating the important role of xanthophyll-dependent photoprotection [33]. Thus, coordinated redox regulation, maintenance of photosynthetic function, and photoprotective responses may represent important components of Cu tolerance.

Although plants possess several mechanisms for coping with excess Cu, their effectiveness differs among species and genotypes [34,35]. This genotypic variation is particularly important in agricultural environments where Cu may accumulate gradually as a consequence of repeated fungicide applications. Knowledge of Cu-tolerant plants can contribute to phytoremediation strategies, site restoration, risk reduction and the selection of suitable plant–microbe combinations, as well as to breeding programmes [36]. In recent years, there has been a strong focus on sustainable agriculture and the transformation of conventional agricultural practices into more sustainable ones [37]. One goal of sustainable agriculture is also the appropriate use and even remediation of soils contaminated with heavy metals [38]. One of the main characteristics of sustainable agriculture is multi-cropping systems. Multi-cropping systems could increase crop diversity and avoid vulnerability to biotic stress, as well as ensure the sustainability of agriculture and increase crop and food production while reducing land use [39]. The unique approach known as the ‘La Piantata Veneta’ is a multi-cropping system originating from Italy and includes grapevines, a supportive tree species (usually mulberry) and a ground-level crop [40]. This system is of particular interest because vineyards are among the agricultural areas where Cu can accumulate as a result of repeated fungicide treatments [41,42]. Mulberry is particularly interesting in this context because of its economic importance, broad range of uses and reported tolerance to heavy metals [43,44,45,46,47,48].

*Morus alba* L. and *M. indica* L. are among the most commonly cultivated mulberry species for sericulture [43,44], while mulberry is also used for fruit, wood, fodder, fibre, tea and medicinal products [45,46,47]. Furthermore, mulberry has shown tolerance to heavy metals in contaminated soil and can contribute to improved soil microbial diversity [48]. The considerable genetic diversity among mulberry genotypes, related in part to their complex reproductive biology and history of selection for sericulture, provides an opportunity to compare varieties with contrasting responses to environmental stress [49]. The present study therefore aimed to assess the tolerance of five mulberry varieties belonging to *M. alba* and *M. indica* to Cu-induced stress in a pot experiment. We evaluated shoot growth, leaf Cu accumulation, the ASC–GSH redox system, total phenolic compounds, photosynthetic pigments, xanthophyll-cycle pigments and maximum photochemical efficiency of PSII (Fv/Fm). Particular emphasis was placed on the relationship between antioxidant and redox responses and the maintenance of photosynthetic function under Cu stress. We hypothesized that Cu-tolerant mulberry varieties would exhibit more effective redox regulation and stronger photoprotective responses than Cu-sensitive varieties. The study further aimed to identify varieties with greater Cu tolerance and potential for cultivation in Cu-contaminated soils.

## 2. Results

### 2.1. Influence of CuSO_4_ Treatment on Plant Development

**Figure 1:**
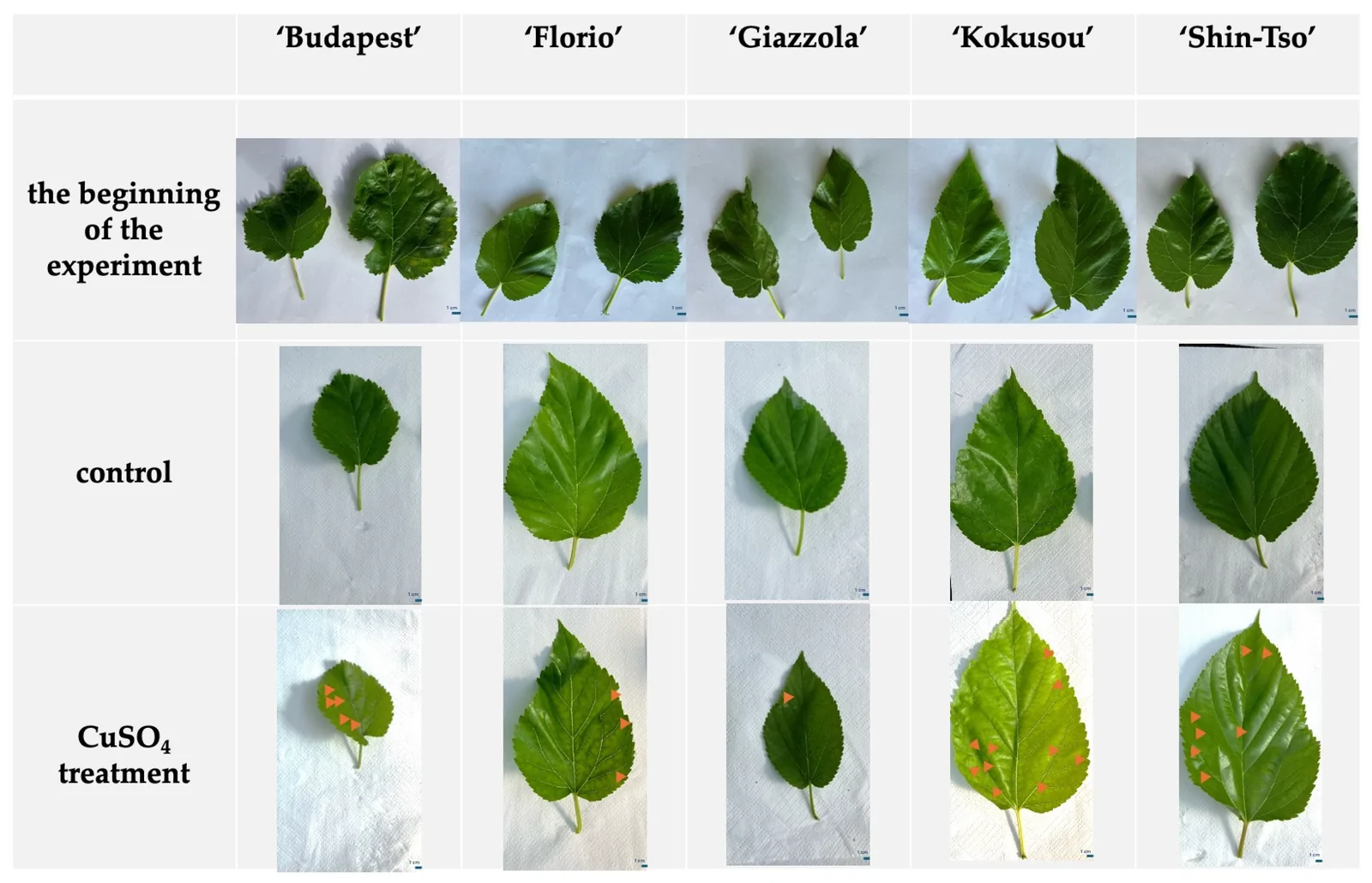
Morphologically visible symptoms of the leaves of different *M. alba* and *M. indica* varieties treated with CuSO_4_ compared to leaves of plants cultivated under control conditions at the beginning of the experiment and after 21 days of CuSO_4_ treatment. Orange arrowheads indicate the chlorotic spots. At the beginning of the experiment, no symptoms were present on the leaves of any of the plants (the leaves on the left side belong to the control plant, and the leaves on the right side to the plant treated with CuSO_4_).

Morphologically visible symptoms on leaves of container-grown two-year old trees treated with CuSO_4_ were visible after 21 days of the experiment (Figure 1). On day 0 of the experiment, there were no visible differences between the leaves of plants treated with CuSO_4_ and plants exposed to control conditions in any of the variety included in the experiment. However, after 21 days from the beginning of the experiment, chlorosis appeared on the leaves of all plants of all varieties included in the experiment treated with CuSO_4_. However, the leaves of the variety ‘Kokusou’ were the most affected. Almost the entire leaves were yellow and had many chlorotic areas towards the leaf margin. Similar symptoms were also observed for the leaves of the local variety ‘Budapest’ and commercial variety ‘Shin-Tso’, but the leaves of these two varieties were less yellowish and developed a smaller number of chlorotic areas. The leaves of the ‘Florio’ and ‘Giazzola’ varieties were more greenish and did not differ significantly from the leaves of plants cultivated under control conditions. They developed a smaller number of individual chlorotic spots near the leaf margin. No other visible symptoms, such as leaf necrosis, leaf deformations or leaf asymmetry, were observed between the treated trees and control trees after 21 days of the experiment.

### 2.2. Influence of CuSO_4_ Treatment on Shoot-Growth Rate

**Figure 2:**
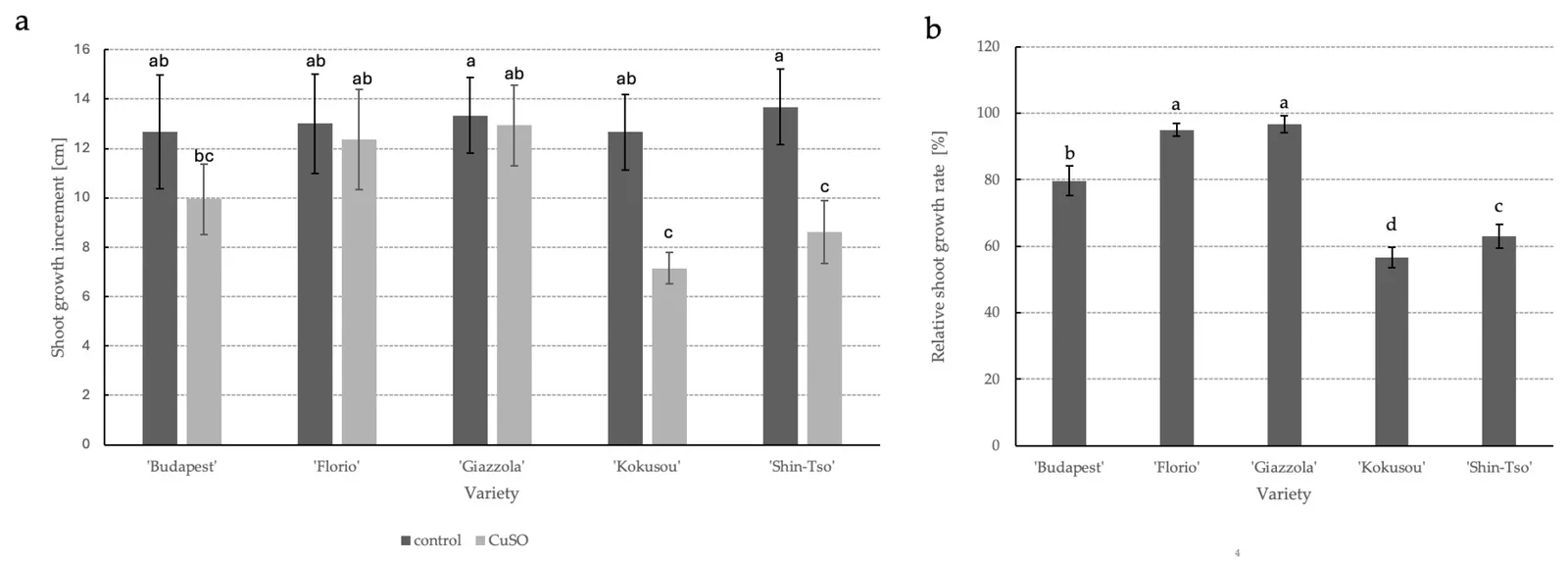
(**a**) The effect of CuSO_4_ treatment on the shoot-growth increment (cm; ±standard deviation, (SD)) of different *M. alba* and *M. indica* varieties 21 days after treatment. Significant differences between the average shoot-growth increment values are indicated by different letters (a–c; post hoc Duncan test, *p* < 0.05, *n* = 30). (**b**) The effect of CuSO_4_ treatment on the relative shoot growth (%; ± SD) of different *M. alba* and *M. indica* varieties 21 days after treatment. Significant differences are indicated by different letters (post hoc Duncan test, *p* < 0.05, *n* = 30).

The mean shoot-growth increment (Figure 2a) and relative shoot-growth rate (Figure 2b) of all five varieties of *M. alba* and *M. indica* included in the experiment treated with CuSO_4_ were lower than those of controls after 21 days of the experiment. However, the relative shoot-growth rate was statistically significantly different among almost all varieties included in the experiment (Figure 2a), so CuSO_4_ treatment inhibited shoot growth to varying degrees in different mulberry varieties. CuSO_4_ treatment least inhibited shoot growth statistically significantly in the varieties ‘Giazzola’ (96.67%) and ‘Florio’ (95.00%), while it most inhibited shoot growth statistically significantly in the variety ‘Kokusou’ (56.67%).

### 2.3. Influence of CuSO_4_ Treatment on Cu Content in Leaves of Different Mulberry Varieties

**Figure 3:**
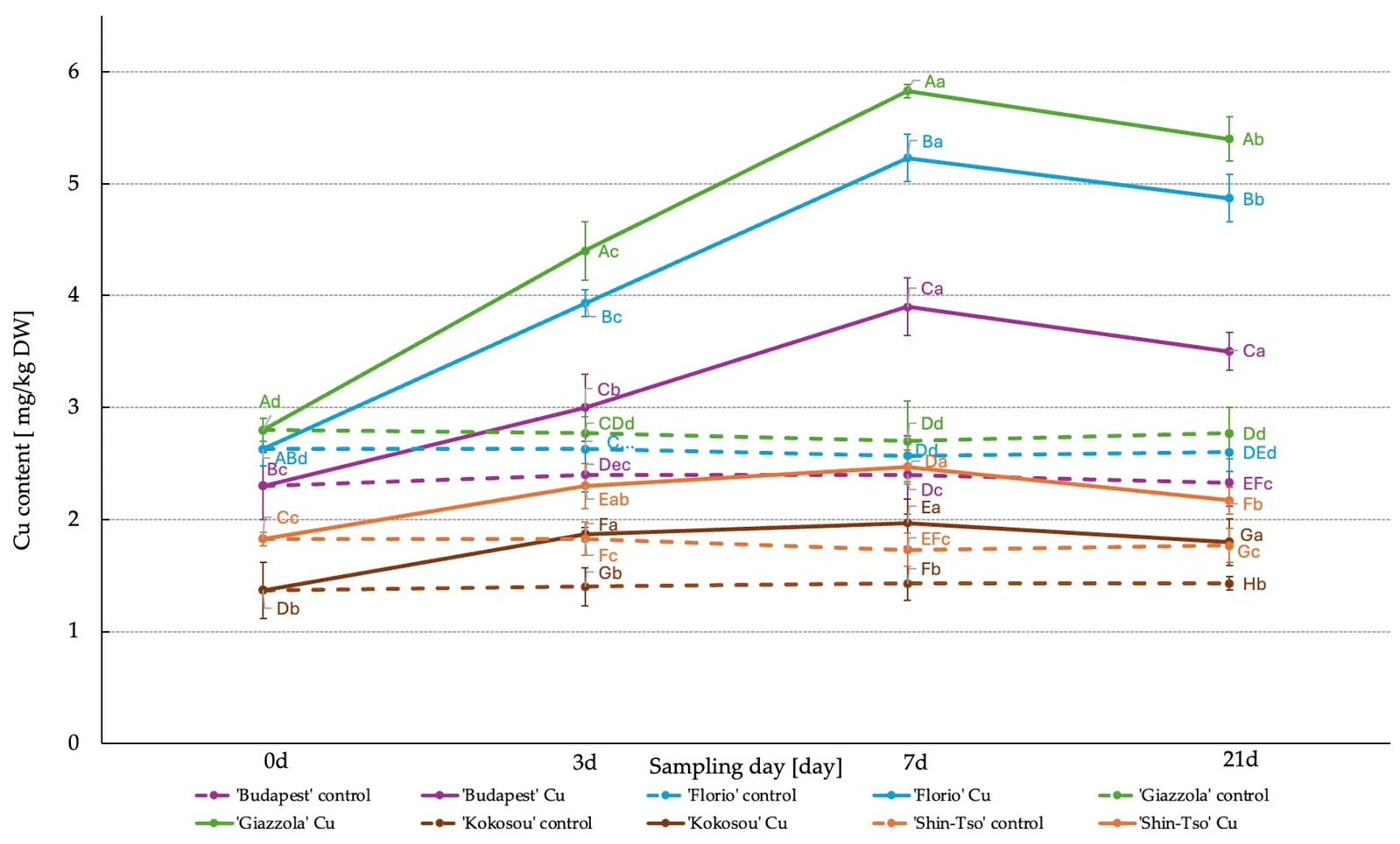
Mean Cu content (mg Cu/kg DW; ±SD) in leaves of *M. alba* varieties ‘Giazzola’, ‘Florio’, ‘Budapest’ and *M. indica* varieties ‘Kokusou ‘and ‘Shin-Tso’ measured at 0, 3, 7 and 21 days of the experiment (*n* = 120). Different lowercase letters (a–e) indicate significant differences among sampling dates within the same variety (*p* < 0.05), as determined using Duncan’s post hoc multiple-range test. Different uppercase letters (A–H) indicate significant differences (*p* < 0.05), determined by the post hoc Duncan test for comparing Cu content in leaves of mulberry plants of different varieties sampled at the same sampling date.

The Cu content in leaves differed statistically significantly between varieties of *M. alba* and *M. indica* cultivated under control conditions, ranging from 1.37 mg/kg DW to 2.80 mg/kg DW, and among varieties treated with CuSO_4_, ranging from 1.37 mg/kg DW to 5.83 mg/kg DW. Statistically significant differences in Cu content were also determined in leaves of CuSO_4_-treated trees of the same varieties sampled at different sampling dates, while in leaves of the same variety cultivated under control conditions there were no statistically significant differences in Cu content between different sampling dates. Under control conditions, leaves of the variety ‘Giazzola’ contained the statistically significantly highest Cu content (2.80 mg/kg DW), while the leaves of ‘Kokusou’ variety contained the statistically significantly lowest (1.37 mg/kg DW) Cu. The Cu content in the leaves of the varieties ‘Budapest’, ‘Florio’ and ‘Giazzola’ treated with CuSO_4_ followed the same trend, reaching the highest levels 7 days (‘Budapest’: 3.90 mg/kg DW; ‘Florio’: 5.23 mg/kg DW; ‘Giazzola’: 5.83 mg/kg DW) after the beginning of the experiment, followed by those measured on the 21st day and 3rd day of the experiment. The lowest contents were measured at the beginning of the experiment. In the leaves of the *M. indica* varieties ‘Kokusou’ and ‘Shin-Tso’ treated with CuSO_4_, the Cu content was the highest after 7 days of treatment (‘Kokusou’:1.97 mg/kg DW;’Shin-Tso’:2.47 mg/kg DW), followed by those after 3 days and 21 days of the experiment, whereas the lowest were analyzed on the 0th day of experiment. More detailed information on the Cu content in the leaves of different varieties sampled at different sampling dates is presented in Figure 3.

### 2.4. Influence of CuSO_4_ Treatment on Total Ascorbate Content and Its Redox Status in Leaves of Different M. alba and M. indica Varieties

**Figure 4:**
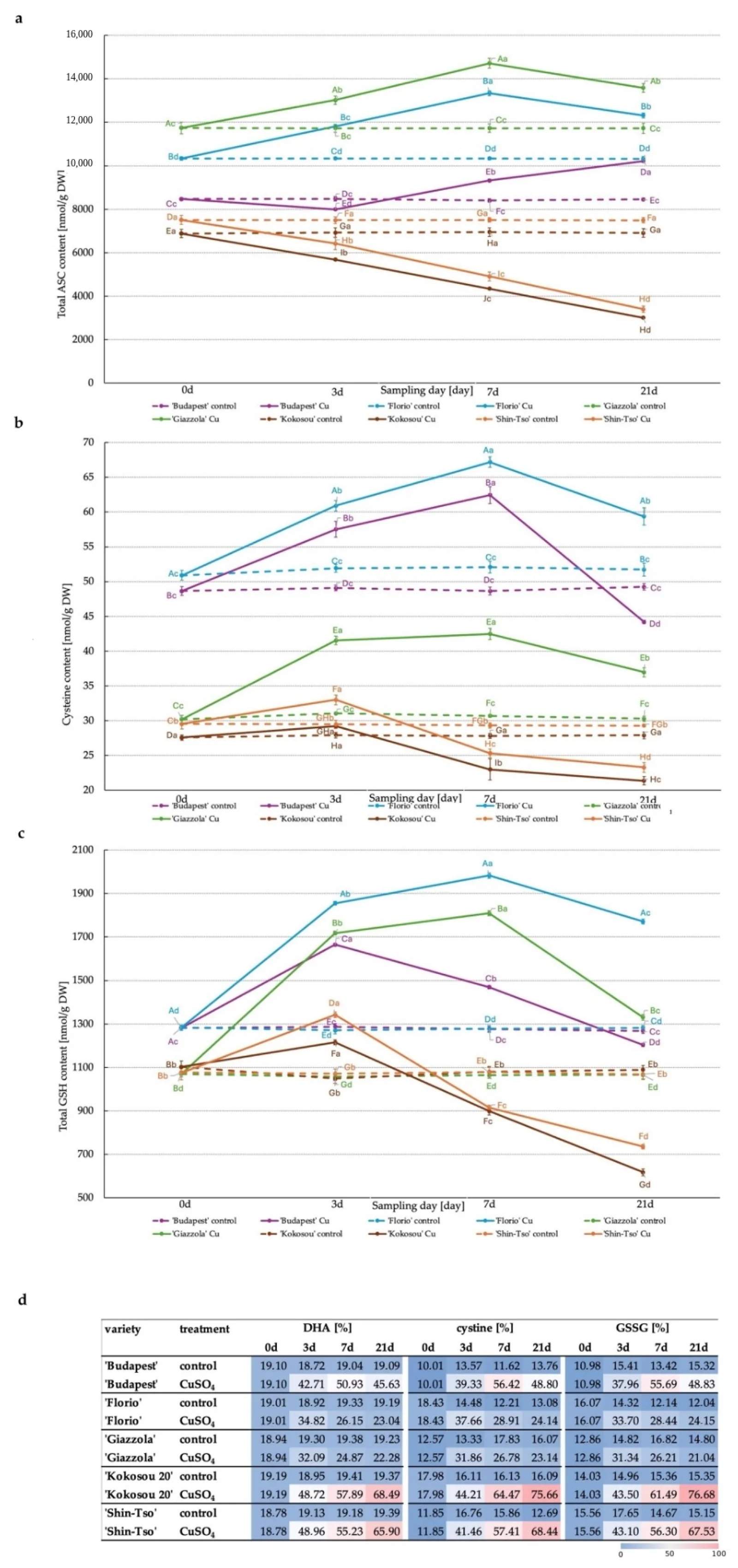
Mean content of (**a**) total ASC (nmol/g DW; ±SD), (**b**) mean content of cysteine (nmol/g DW; ±SD) and (**c**) mean content of total GSH (nmol/g DW; ±SD) in leaves of the varieties of *M. alba* and *M. indica* included in the experiment at 0, 3, 7 and 21 days of the experiment (*n* = 120). Different lowercase letters (a–d) indicate significant differences (*p* < 0.05), determined by the post hoc Duncan test for comparing total ASC content, cysteine content and total GSH content in leaves of the same variety sampled at different sampling dates. Different uppercase letters (A–J) indicate significant differences (*p* < 0.05), determined by the post hoc Duncan test for comparing total ASC content, cysteine content and total GSH content in leaves of different mulberry varieties sampled at the same sampling date. (**d**) Heat map showing the change in mean values of DHA ratio (%), cystine ratio (%) and GSSG ratio (%) in leaves of the varieties of *M. alba* and *M. indica* included in the experiment at 0, 3, 7 and 21 days of the experiment (*n* = 120).

The total ASC content in leaves differed statistically significantly between individual varieties of *M. alba* and *M. indica* cultivated under control conditions (6883.72–11,742.91 nmol/g DW) and between varieties treated with CuSO_4_ (3009.50–14,716.80 nmol/g DW). Statistically significant differences in the total ASC content were also determined in leaves of CuSO_4_-treated plants of the same variety sampled at different sampling dates. In contrast, no statistically significant differences among sampling dates were observed in leaves of control plants of the corresponding variety. Under control conditions, the leaves of the variety ‘Giazzola’ contained, statistically significantly, the highest total ASC content (11,742.91 nmol/g DW), while the leaves of ‘Kokusou’ contained, statistically significantly, the lowest total ASC content (6883.73 nmol/g DW). The temporal pattern of total ASC content across sampling dates differed among CuSO_4_-treated varieties. In the local variety ‘Budapest’, the highest content was reached on the 21st day (10,212.60 nmol/g DW) of the experiment, followed by the 7th day and the 0th day of the experiment, while the lowest content was determined on the 3rd day of the experiment. Total ASC content in the ‘Florio’ and ‘Giazzola’ varieties peaked on the 7th day of the experiment, reaching 13,339.90 nmol/g DW and 14,716.80 nmol/g DW, respectively. This was followed by the 21st day of the experiment and the 3rd day of the experiment. In the ‘Kokusou’ and ‘Shin-Tso’ varieties, the content of total ASC decreased throughout the experiment, being the highest on the 0th day (‘Kokusou’: 6883.72 nmol/g DW; ‘Shin-Tso’: 7505.43 nmol/g DW) of the experiment and the lowest on the 21st day of the experiment. For each variety, the DHA ratio differed significantly between the leaves of control and CuSO_4_-treated plants. Statistically significant differences in the DHA ratio were observed among the CuSO_4_-treated varieties, with values ranging from 18.78% to 68.49%. In contrast, no statistically significant differences were detected among varieties grown under control conditions, in which the DHA ratio remained below 20%. The DHA ratio also differed statistically significantly in leaves of varieties treated with CuSO_4_ between individual sampling dates. The lowest values in all CuSO_4_-treated varieties were determined at the beginning of the experiment. Among the treated plants, the highest DHA ratio (68.49%) was determined in leaves of the ‘Kokusou’ variety sampled on the 21st day of the experiment. In the local variety ‘Budapest’, the highest DHA ratio (50.93%) was reached on the 7th day of the experiment, followed by the 21st day of the experiment. In the ‘Florio’ and ‘Giazzola’ varieties, the highest ratio of DHA was determined on the 3rd day of the experiment, reaching 34.82% and 32.09% respectively. This was followed by the 7th day of the experiment and the 21st day of the experiment. In the ‘Kokusou’ and ‘Shin-Tso’ varieties, the increase was gradual, reaching the highest values on the 21st day (‘Kokusou’: 68.49%; ‘Shin-Tso’: 65.90%). More detailed data on the content of total ASC and DHA ratio in leaves of different varieties of *M. alba* and *M. indica* included in the experiment are presented in Figure 4a,d and Supplementary Table S1.

### 2.5. Influence of CuSO_4_ Treatment on the Content of Low-Molecular-Weight Thiols and Their Redox Status in Leaves of Different M. alba and M. indica Varieties

The cysteine content in leaves of *M. alba* and *M. indica* varieties included in the experiment differed statistically significantly between different varieties cultivated under control conditions (27.57–52.08 nmol/g DW) and those treated with CuSO_4_ (21.37–67.17 nmol/g DW). Statistically significant differences in cysteine content were observed among sampling dates in leaves of CuSO_4_-treated plants within the same variety. In contrast, no statistically significant differences among sampling dates were detected in leaves of control plants of the corresponding variety. Under control conditions, the leaves of the variety ‘Florio’ contained the statistically significantly highest cysteine content (52.08 nmol/g DW), while the leaves of ‘Kokusou’ contained the statistically significantly lowest (27.57 nmol/g DW) cysteine content. The temporal pattern of cysteine content across sampling dates varied among the CuSO_4_-treated varieties. In the local variety ‘Budapest’, the highest cysteine content (62.44 nmol/g DW) was reached on the 7th day of the experiment, followed by the 3rd day and the 0th day of the experiment, while the lowest cysteine content was determined on the 21st day of the experiment. In the ‘Florio’ and ‘Giazzola’ varieties, the highest cysteine content was determined on the 7th day of the experiment, reaching 67.17 nmol/g DW and 42.49 nmol/g DW respectively. This was followed by the 3rd day of the experiment, the 21st day of the experiment and the 0th day of the experiment. In the ‘Kokusou’ and ‘Shin-Tso’ varieties, the highest content of cysteine was determined on day 3 of the experiment (‘Kokusou’: 29.23 nmol/g DW; ‘Shin-Tso’: 33.00 nmol/g DW), followed by the 0th day, 7th day and 21st day of the experiment. For each variety, the cystine ratio differed significantly between the leaves of control plants and those of CuSO_4_-treated plants. Statistically significant differences in the cystine ratio were also observed among the CuSO_4_-treated varieties, with values ranging from 10.01% to 75.66%. In contrast, no statistically significant differences were detected among varieties grown under control conditions, in which the cystine ratio ranged from 10.01% to 18.43%. The cystine ratio also differed statistically significantly in leaves of plants of the same variety treated with CuSO_4_ between different sampling dates. Among the treated plants, the highest cystine ratio (75.66%) was determined in leaves of the ‘Kokusou’ variety sampled on the 21st day of the experiment and the lowest in leaves of the ‘Budapest’ variety sampled on the 0th day of the experiment. The changing trend of cystine ratio between individual sampling dates in plants treated with CuSO_4_ differed between varieties. In the ‘Budapest’ variety, the highest ratio (56.42%) was reached on the 7th day of the experiment, followed by the 21st day of the experiment and the 3rd day of the experiment, while the lowest was determined on the 0th day of the experiment. In the ‘Florio’ and ‘Giazzola’ varieties, the highest ratio of cystine was determined on the 3rd day of the experiment, reaching 37.66% and 31.86%, respectively. This was followed by the 7th day of the experiment, the 21st day of the experiment and the 0th day of the experiment. In the ‘Kokusou’ and ‘Shin-Tso’ varieties, the cystine ratio in the leaves increased throughout the experiment—the highest was on the 21st day (‘Kokusou’: 75.66%; ‘Shin-Tso’: 68.44%) and the lowest was on the 0th day of the experiment. More detailed data on the cysteine content and cystine ratio in leaves of different varieties included in the experiment are presented in Figure 4b,d and Supplementary Table S1.

The total GSH content in leaves of *M. alba* and *M. indica* varieties included in the experiment differed statistically significantly between different varieties cultivated under control conditions (1046.48–1287.06 nmol/g DW) and between different varieties treated with CuSO_4_ (617.12–1982.28 nmol/g DW). Statistically significant differences in total GSH content were also present in the leaves of CuSO_4_-treated plants of the same varieties sampled at different sampling dates. In contrast, no statistically significant differences among sampling dates were found in leaves of the same variety cultivated under control conditions. Under control conditions, the leaves of the local variety ‘Budapest’ contained, statistically significantly, the highest total GSH content (1287.06 nmol/g DW) and the leaves of the variety ‘Kokusou’ contained the lowest (1046.48 nmol/g DW) total GSH content. In the ‘Budapest’ variety, the highest content (1664.43 nmol/g DW) was reached on the 3rd day of the experiment, followed by the 7th day of the experiment and the 0th day of the experiment, while the lowest content was determined on the 21st day of the experiment. In the ‘Florio’ and ‘Giazzola’ varieties, the highest total GSH content was determined on the 7th day of the experiment, reaching 1809.28 nmol/g DW and 1982.28 nmol/g DW, respectively. This was followed by the 3rd day of the experiment, the 21st day of the experiment and the 0th day of the experiment. In the ‘Kokusou’ and ‘Shin-Tso’ varieties, the highest total GSH content was determined on the 3rd day of the experiment (‘Kokusou’: 1215.21 nmol/g DW; ‘Shin-Tso’: 1341.75 nmol/g DW), followed by the 0th day, 7th day and 21st day of the experiment. The GSSG ratio in leaves of different varieties differed statistically significantly within each variety between the leaves of plants cultivated under control conditions and those treated with CuSO_4_. Statistically significant differences were also determined in the GSSG ratio in leaves of different varieties treated with CuSO_4_ (10.98–76.68%), while there were no statistically significant differences in the GSSG ratio in leaves of plants cultivated under control conditions (bellow 18%). The GSSG ratio also differed statistically significantly in leaves of plants treated with CuSO_4_ between different sampling dates. Among the treated plants, the highest GSSG ratio (76.68%) was determined in leaves of the ‘Kokusou’ variety sampled on the 21st day of the experiment and the lowest (10.98%) in leaves of the ‘Budapest’ local variety sampled on day 0 of the experiment. The changing trend of GSSG ratio between individual sampling dates in plants treated with CuSO_4_ differed between varieties. In the ‘Budapest’ variety, the highest GSSG ratio (55.69%) was reached on the 7th day of the experiment, followed by the 21st day of the experiment and the 3rd day of the experiment, while the lowest was determined on the 0th day of the experiment. In the ‘Florio’ (33.70%) and ‘Giazzola’ (31.34%) varieties, the highest GSSG ratio was determined on the 3rd day of the experiment, followed by the 7th day of the experiment, the 21st day of the experiment and the 0th day of the experiment. In the leaves of the ‘Kokusou’ and ‘Shin-Tso’ varieties, the GSSG ratio increased throughout the experiment—the highest was on the 21st day of the experiment (‘Kokusou’: 76.68%; ‘Shin-Tso’: 67.53%) and the lowest on the 0th day of the experiment. More detailed data on the content of total GSH and GSSG ratio in leaves of different *M. alba* and *M. indica* varieties are presented in Figure 4c,d and Supplementary Table S1.

### 2.6. Influence of CuSO_4_ Treatment on the Total Phenolic Content in Leaves of Different M. alba and M. indica Varieties

**Figure 5:**
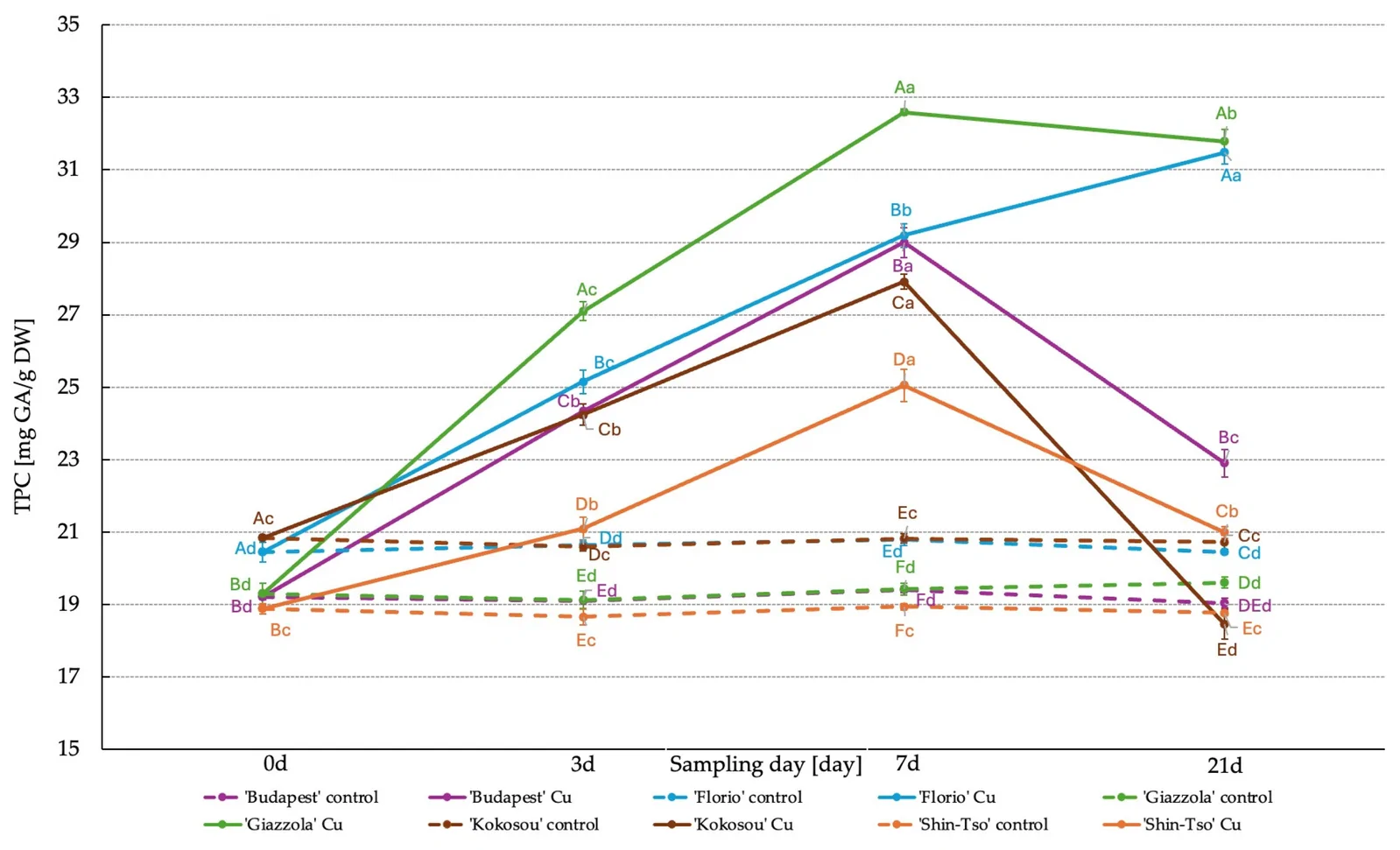
Mean TPC (mg GA/g DW; ±SD) in leaves of the varieties of *M. alba* and *M. indica* included in the experiment at 0, 3, 7 and 21 days of the experiment (*n* = 120). Different lowercase letters (a–d) indicate significant differences (*p* < 0.05), determined by the post hoc Duncan test for comparing TPC in leaves of the same variety sampled at different sampling dates. Different uppercase letters (A–F) indicate significant differences (*p* < 0.05), determined by the post hoc Duncan test for comparing TPC in leaves of different varieties sampled at the same sampling date.

The TPC in leaves of *M. alba* and *M. indica* varieties included in the experiment differed statistically significantly between different varieties cultivated under control conditions and between different varieties treated with CuSO_4_. Statistically significant differences in TPC were also present in the leaves of CuSO_4_-treated plants of the same variety sampled at different sampling dates, while in leaves of the same variety cultivated under control conditions, there were no statistically significant differences in the TPC between different sampling dates. Under control conditions, the leaves of the variety ‘Kokusou’ contained the highest TPC (20.84 mg GA/g DW) and the leaves of the variety ‘Shin-Tso’ contained the lowest TPC (18.66 mg GA/g DW). The changing trend of TPC between individual sampling dates in plants treated with CuSO_4_ differed between different varieties. In the local variety ‘Budapest’ and variety ‘Shin-Tso’, the highest TPC was reached on the 7th day of the experiment (‘Budapest’: 28.99 mg GA/g DW; ‘Shin-Tso’: 25.05 mg GA/g DW), followed by the 3rd day and the 21st day of the experiment, while the lowest TPC for both varieties was determined on the 0th day of the experiment. In the ‘Florio’ variety, TPC increased throughout the experiment—the highest (31.48 mg GA/g DW) was determined on the 21st day and the lowest on the 0th day of the experiment. In the ‘Giazzola’ variety, the highest TPC (32.59 mg GA/g DW) was determined on the 7th day of the experiment, followed by the 21st day, the 3rd day and the 0th day of the experiment. In the ‘Kokusou’, the highest TPC was determined on the 7th day of the experiment (27.91 mg GA/g DW), followed by the 3rd day, 0th day and 21st day of the experiment. More detailed data on the TPC in leaves of different *M. alba* and *M. indica* varieties are presented in Figure 5.

### 2.7. Influence of CuSO4 Treatment on the Photosynthetic Pigments in Leaves of Different M. alba and M. indica Varieties

**Figure 6:**
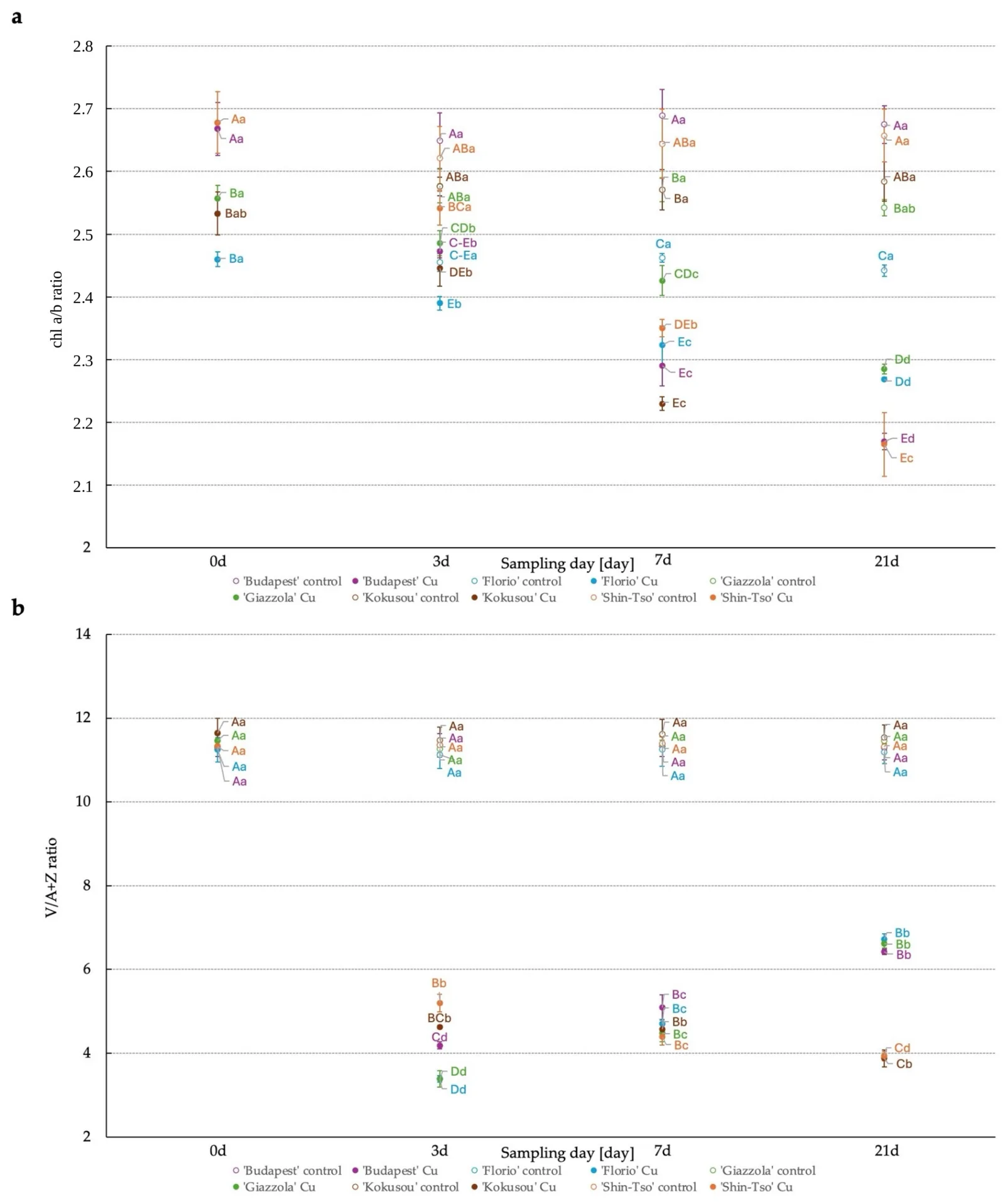
Mean (**a**) chl a/b ratio (±SD) and mean (**b**) V/A+Z ratio (±SD) in leaves of *M. alba* and *M. indica* varieties at the 0th, 3rd, 7th and 21st days of the experiment (*n* = 120). Different lowercase letters (a–d) indicate significant differences (*p* < 0.05), determined by the post hoc Duncan test for comparing chl a/b ratio and V/A+Z ratio in leaves of the same variety sampled at different sampling dates. Different uppercase letters (A–E) indicate significant differences (*p* < 0.05), determined by the post hoc Duncan test for comparing chl a/b ratio and V/A+Z ratio in leaves of different varieties sampled at the same sampling date.

The content of chl a, chl b, total carotenoids and the ratio between chl a and chl b contents (chl a/b) in leaves differed statistically significantly between different varieties cultivated under control conditions and between different varieties treated with CuSO_4_. Statistically significant differences in the aforementioned compound content were also present in leaves of CuSO_4_-treated plants of the same variety sampled at different dates, while in leaves of the same variety cultivated under control conditions there were no statistically significant differences. Under control conditions the highest chl a content was contained in the leaves of the ‘Budapest’ variety (4.059 mg GA/g DW), while the leaves of the variety ‘Kokusou’ contained the highest chl b content (1.573 mg/g DW), and the leaves of the variety ‘Shin-Tso’ contained the highest chl a/b (2.678) and also the highest total carotenoid content (0.928 mg/g DW). In contrast, the leaves of ‘Florio’ cultivated under control conditions contained the lowest chl a content (3.788 mg/g DW) and the lowest chl a/b (2.442), the leaves of ‘Shin-Tso’ contained the lowest chl b content (1.502 mg/g DW), and the leaves of ‘Kokusou’ contained the lowest total carotenoid content (0.880 mg/g DW). The temporal trend of chl a and chl b content, as well as chl a/b between individual sampling dates in plants treated with CuSO_4,_ was the same for all varieties included in the experiment. Their values decreased throughout the experiment—the highest contents were determined on the 0th day and the lowest on the 21st day of the experiment. The temporal trend of total carotenoid content in leaves between individual sampling dates in plants treated with CuSO_4_ differed between varieties. In the local variety ‘Budapest’, the highest total carotenoids content (0.967 mg/g DW) was determined on the 3rd day of the experiment, followed by the 7th day of the experiment and the 0th day of the experiment, while the lowest total carotenoid content was determined on the 21st day of the experiment. In the ‘Florio’ (1.190 mg/g DW) and ‘Giazzola’ (1.169 mg/g DW) varieties, the highest total carotenoid content was determined on the 7th day of the experiment, followed by the 3rd day, the 21st day and the 0th day of the experiment. In the ‘Kokusou’ and ‘Shin-Tso’ varieties, the total carotenoid content decreased throughout the experiment—the highest was on the 0th day of the experiment (‘Kokusou’: 0.880 mg/g DW; ‘Shin-Tso’: 0.928 mg/g DW) and the lowest on the 21st day of the experiment. More detailed data on the chl a and chl b content, chl a/b, and the total carotenoid content in leaves of different *M. alba* and *M. indica* varieties are presented in Table 1 and Figure 6a.

The content of neoxanthin and lutein in leaves of *M. alba* and *M. indica* varieties differed statistically significantly between different varieties cultivated under control conditions and between those treated with CuSO_4_, while the violaxanthin content and the total content of antheraxanthin and zeaxanthin (A+Z), as well as the ratio between the violaxanthin content and the sum of antheraxanthin and zeaxanthin content (V/A+Z), differed statistically significantly only in the leaves of plants of different varieties treated with CuSO_4_. Statistically significant differences in the aforementioned xanthophyll pigments’ content were also present in leaves of CuSO_4_-treated plants of the same varieties sampled at different sampling dates, while in leaves of plants of the same variety cultivated under control conditions there were no statistically significant differences in their content between different sampling dates. Under control conditions the highest neoxanthin content was determined in leaves of ‘Florio’ (47.4 µg/g DW). The leaves of the local variety ‘Budapest’ were characterized by the highest lutein content (0.367 µg/g DW), while ‘Kokusou’ contained the highest violaxanthin content (39.3 µg/g DW). In contrast, the lowest neoxanthin content (44.5 µg/g DW) was determined in the variety ‘Kokusou’, the leaves of ‘Giazzola’ had the lowest lutein content (348.2 µg/g DW), and the leaves of ‘Florio’ had the lowest violaxanthin content (37.7 µg/g DW). The A+Z in the leaves of all varieties grown under control conditions was around 3.4 µg/g DW. The temporal pattern of neoxanthin content across sampling dates varied among the CuSO_4_-treated varieties. In the ‘Budapest’, ‘Kokusou’ and ‘Shin-Tso’ varieties, the neoxanthin content in the leaves decreased throughout the experiment—the highest was on the 0th day (‘Budapest’: 45.9 µg/g DW; ‘Kokusou’: 44.5 µg/g DW; ‘Shin-Tso’: 46.3 µg/g DW) and the lowest on the 21st day of the experiment. In the ‘Florio’ variety the highest neoxanthin content (52.0 µg/g DW) was determined on the 7th day of the experiment, followed by the 21st day, the 3rd day and the 0th day of the experiment. In the ‘Giazzola’ variety the highest neoxanthin content (50.3 µg/g DW) was determined on the 3rd day of the experiment, followed by the 7th day, the 21st day and the 0th day of the experiment.

A similar temporal trend between individual sampling dates in plants treated with CuSO_4_ was determined for lutein. In the local variety ‘Budapest’, the highest lutein content (383.6 µg/g DW) was reached on the 7th day of the experiment, followed by the 3rd day of the experiment and the 0th day of the experiment, while the lowest was determined on the 21st day of the experiment. In the ‘Florio’ variety the highest lutein content (452.8 µg/g DW) was determined on the 7th day, followed by the 21st day, the 3rd day and the 0th day of the experiment. In the ‘Giazzola’ variety the highest lutein content (455.6 µg/g DW) was determined on the 7th day, followed by the 3rd day, the 21st day and the 0th day of the experiment. In the ‘Kokusou’ and ‘Shin-Tso’ varieties the lutein content decreased throughout the experiment—the highest was on the 0th day of the experiment (‘Kokusou’: 361.9 µg/g DW; ‘Shin-Tso’: 358.9 µg/g DW) and the lowest on the 21st day of the experiment.

The temporal pattern of violaxanthin content was as follows: in the ‘Budapest’, ‘Kokusou’, and ‘Shin-Tso’ varieties, leaf violaxanthin content decreased throughout the experiment. The highest values were recorded on day 0 (‘Budapest’: 38.4 µg/g DW; ‘Kokusou’: 39.3 mg µg/g DW; ‘Shin-Tso’: 39.0 µg/g DW), whereas the lowest values were observed on day 21. In the ‘Florio’ variety the highest violaxanthin content (43.1 µg/g DW) was determined on the 7th day, followed by the 21st day of the experiment, the 0th day and the 3rd day of the experiment. In the ‘Giazzola’ variety the highest violaxanthin content (40.6 µg/g DW) was determined on the 7th day of the experiment, followed by the 3rd day, the 21st day and the 0th day of the experiment.

The temporal trend of A+Z between individual sampling dates in plants treated with CuSO_4_ was the same for all varieties included in the experiment. The highest contents were determined on the 3rd day (‘Budapest’: 8.3 µg/g DW; ‘Florio’: 11.0 µg/g DW; ‘Giazzola’: 11.7 µg/g DW; ‘Kokusou’: 6.1 µg/g DW; ‘Shin-Tso’: 6.0 µg/g DW), followed by the contents determined on the 7th day, the 21st day and the 0th day of the experiment. On day 0 of the experiment, the A+Z content in the leaves of all varieties was approximately 3.4 µg/g DW. The temporal pattern of the V/(A+Z) ratio across sampling dates varied among the CuSO_4_-treated varieties. In the ‘Budapest’, ‘Florio’ and ‘Giazzola’ varieties, the V/A+Z was highest on the 0th day of the experiment (approx. 11), followed by the 21st day, the 7th day and the 3rd day of the experiment. In the ‘Kokusou’ and ‘Shin-Tso’ varieties, the V/A+Z ratio decreased throughout the experiment, being the highest on the 0th day of the experiment (approx. 11) and the lowest on the 21st day of the experiment. More detailed data on the contents of xanthophyll pigments in leaves of different *M. alba* and *M. indica* varieties are presented in Table 2 and Figure 6b.

### 2.8. Influence of CuSO_4_ Treatment on the Maximum Quantum Yield of Photosystem II in Different M. alba and M. indica Varieties

**Figure 7:**
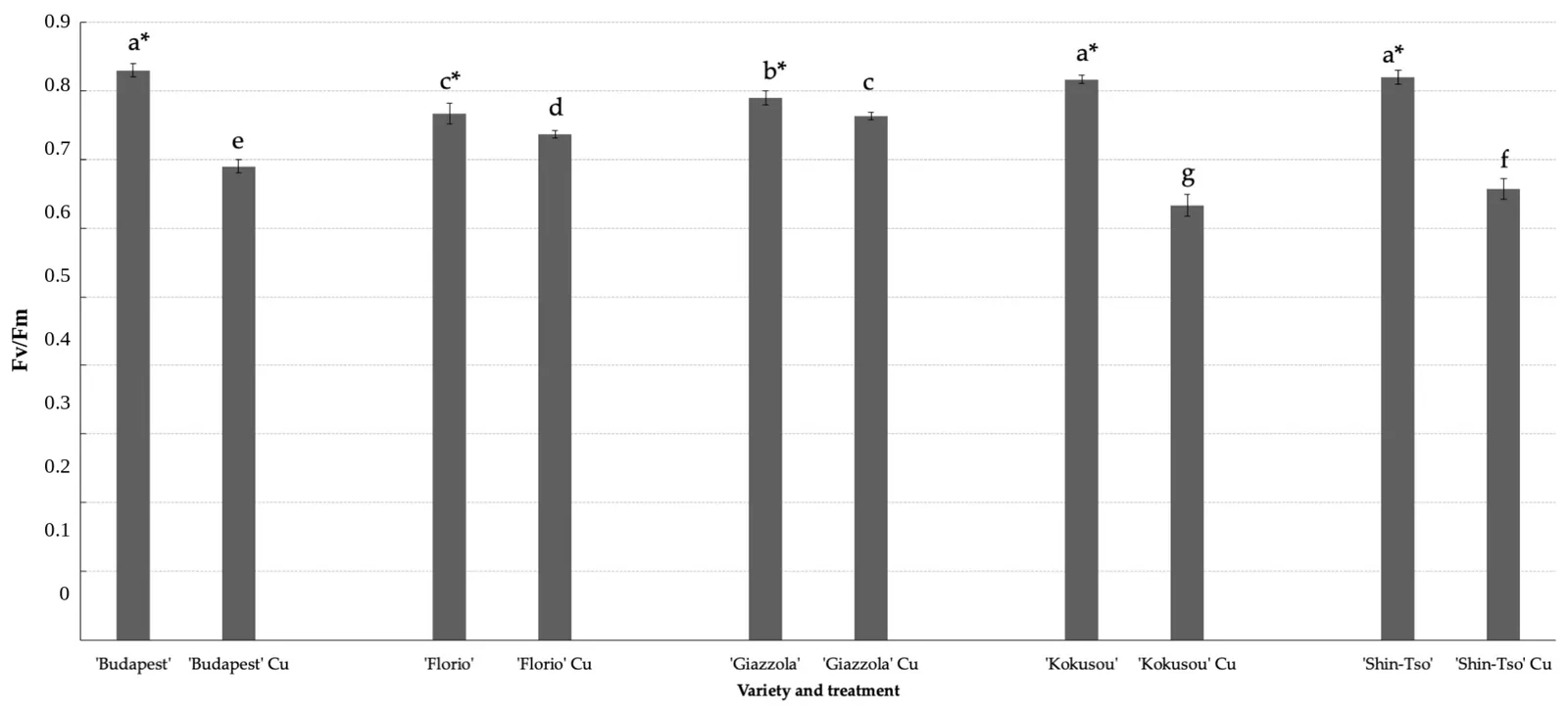
The influence of CuSO_4_ treatment on the maximum quantum yield of PSII (Fv/Fm) ± SD of different *M. alba* and *M. indica* varieties after 21 days of the experiment. Significant differences between the average Fv/Fm values are indicated by different letters (a–g; post hoc Duncan test, *p* < 0.05, *n* = 30). Asterisks indicate a statistically significant higher value between the average values of Fv/Fm of plants of the same variety grown under control conditions and exposed to CuSO_4_ (post hoc Duncan test, *p* < 0.05, *n* = 30).

Monitoring of the maximum quantum yield of PSII revealed that, after 21 days, Fv/Fm values were significantly lower in CuSO_4_-treated plants than in control plants across all examined varieties of *M. alba* and *M. indica* (Figure 7). However, Fv/Fm values also differed statistically significantly between varieties cultivated under control conditions. Among controls, the highest average Fv/Fm values were measured in the local variety ‘Budapest’ (0.83) and the statistically significant lowest values in the variety ‘Florio’. While 21 days after CuSO_4_ exposure, the statistically significant highest Fv/Fm values were determined in the variety ‘Giazzola’ (0.76) and the statistically significant lowest values were determined in the variety ‘Kokusou’.

### 2.9. Influence of CuSO_4_ Treatment, Sampling Time and Variety on the Biochemical Characteristics of M. alba and M. indica Varieties

The effects of CuSO_4_ treatment, sampling time after CuSO_4_ treatment and variety on the biochemical characteristics of leaves of *M. alba* and *M. indica* were further tested with a two-way analysis of variance. Cu content, DHA ratio, cysteine content, cystine ratio, total GSH content, GSSG ratio, TPC, chl a content, chl b content, total carotenoid content, chl a/b and neoxanthin, violaxanthin, antheraxanthin, zeaxanthin and A+Z contents were significantly influenced by variety, CuSO_4_ treatment, sampling date, combination of variety and CuSO_4_ treatment, combination of variety and sampling date, combination of CuSO_4_ treatment and sampling date and combination of variety, CuSO_4_ treatment and sampling date. Furthermore, total ASC content and lutein content were significantly influenced by variety, sampling date, combination of variety and CuSO_4_ treatment, combination of variety and sampling date, combination of CuSO_4_ treatment and sampling date and combination of variety, CuSO_4_ treatment and sampling date. The V/A+Z was significantly influenced by CuSO_4_ treatment, CuSO_4_ sampling time, combination of variety and CuSO_4_ treatment, combination of variety and sampling, combination of variety and CuSO_4_ treatment and sampling date. The results from a two-way analysis of variance are presented in Table S2.

### 2.10. Ward’s Clustering of M. alba and M. indica Varieties According to Their Response to CuSO_4_ Treatment

**Figure 8:**
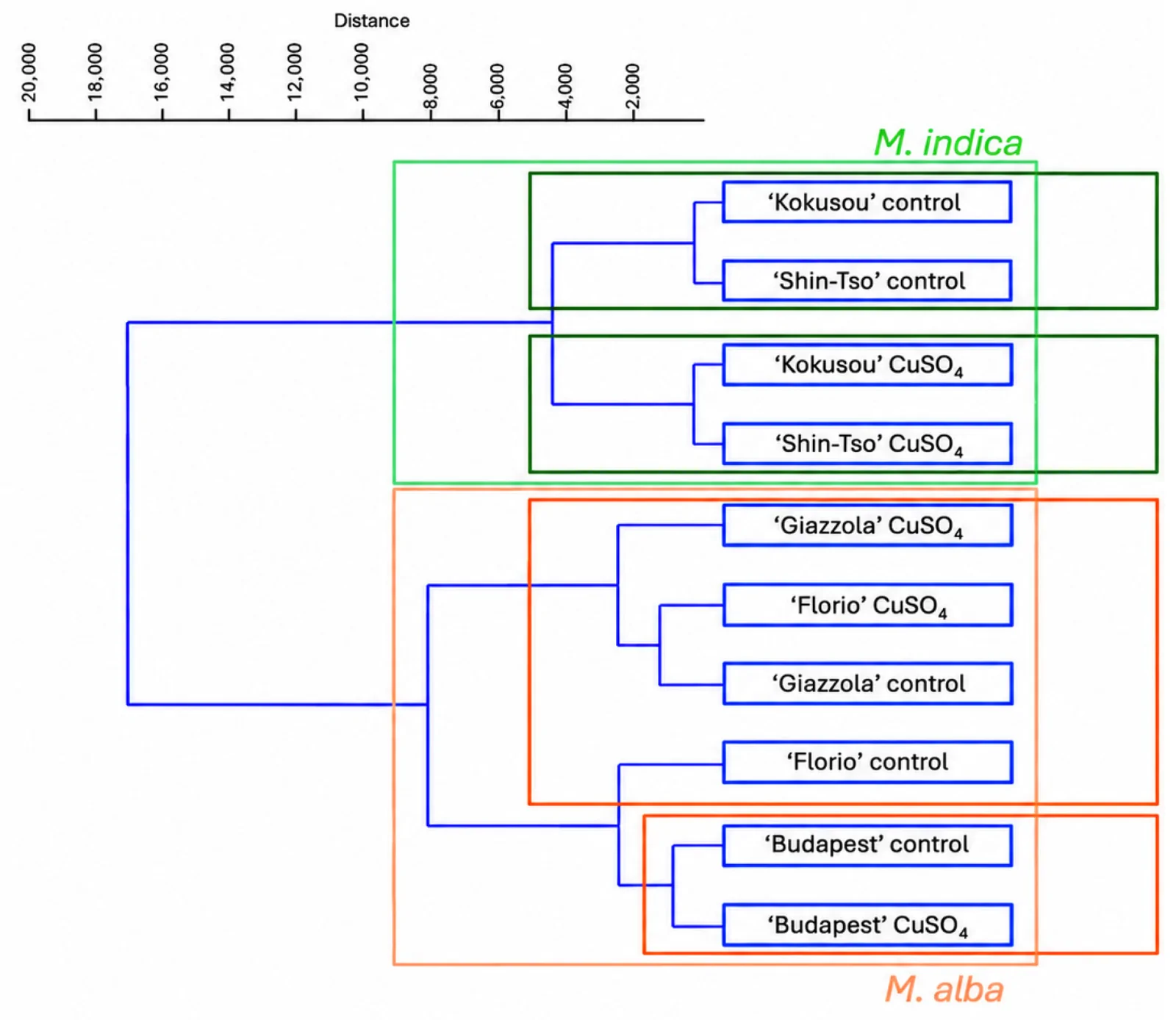
Phylogenetic tree according to Ward (constructed on observed parameters in the experiment) of *M. alba* and *M. indica* varieties included in the experiment, cultivated under control conditions and under Cu-stress conditions.

To visualize similarities in the responses of different varieties to Cu-induced stress based on the measured parameters, a dendrogram was generated using Ward’s hierarchical clustering method. The dendrogram (Figure 8) shows a clear separation into two major clusters at high linkage distance. Cluster I comprised both *M. indica* varieties, ‘Kokusou’ and ‘Shin-Tso’, and included control and CuSO_4_-treated plants. Cluster II comprised all *M. alba* varieties included in the experiment—‘Budapest’, ‘Florio’, and ‘Giazzola’—and included control and CuSO_4_-treated plants. These results indicate that species identity was a stronger determinant of clustering than CuSO_4_ treatment. Intrinsic physiological and biochemical differences between *M. indica* and *M. alba* dominate the dataset—metabolic profiles differ more between species than between stress treatments within species. In Cluster I, there is a division into two subclusters—the first subcluster includes the samples ‘Kokusou’ control and ‘Shin-Tso’ control, and the second includes the samples ‘Kokusou’ CuSO_4_- and ‘Shin-Tso’ CuSO_4_-treated plants. Based on this, it can be concluded that Cu-induced stress induces similar physiological responses in both *M. indica* varieties included in the experiment. In Cluster II, it was observed that the samples ‘Florio’ CuSO_4_, ‘Giazzola’ control and ‘Giazzola’ CuSO_4_ are grouped in one subcluster, while in the second subcluster of Cluster II, ‘Budapest’ control, ‘Budapest’ CuSO_4_ and ‘Florio’ control are grouped. Unlike *M. indica* varieties, clustering in *M. alba* varieties is less strictly separated by treatment. This suggests greater variety-specific variability in Cu response in *M. alba* varieties compared to *M. indica* varieties included in the experiment. This implies that in *M. alba*, variety and treatment effects are more intertwined and that Cu-induced stress induces variety-dependent metabolic response.

### 2.11. Correlations Among Biochemical Parameters Measured in M. alba and M. indica Varieties at Different Sampling Times Following CuSO_4_ Treatment

**Figure 9:**
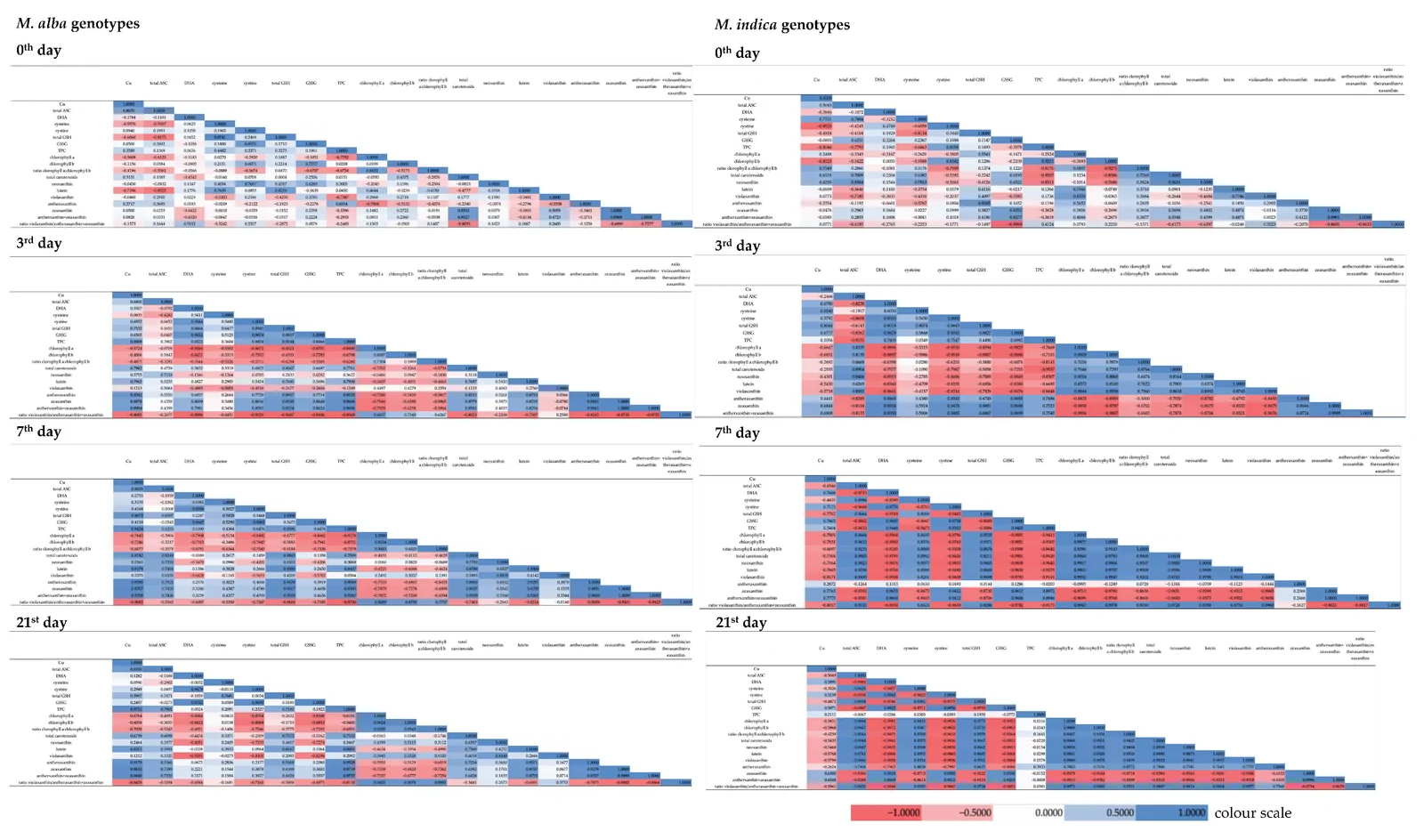
The Pearson correlation heat maps for the relationships between certain metabolites in the leaves of *M. alba* and *M. indica* sampled 0, 3, 7 and 21 days after treatment with CuSO_4._ The matrix displays correlation coefficients (r), with values ranging from −1 (strong negative correlation) to +1 (strong positive correlation), alongside a colour gradient for visual interpretation: blue represents positive correlations, while red indicates negative correlations.

Pearson correlation analysis revealed temporal changes in relationships among measured metabolites in *M. alba* and *M. indica* varieties after Cu exposure (Figure 9). At day 0, several strong correlations (|r| ≥ 0.80) were observed for both species. In *M. alba*, strong negative correlations were observed between total ASC and total GSH, and between ASC and lutein. Strong positive correlations were observed between cysteine and total GSH, and between GSSG and cystine. Additional strong positive correlations were present between lutein and total GSH, as well as within the xanthophyll pigments, including zeaxanthin with total carotenoids, and A+Z with zeaxanthin and total carotenoids. In *M. indica*, numerous strong correlations were analyzed. Strong negative correlations were observed between cystine and Cu, total GSH and cysteine, TPC and Cu, and between chl b and Cu. Strong negative correlations were also present between total carotenoids and TPC, and between chl b and total carotenoids. Strong positive correlations were detected between TPC and cystine, chl b and TPC, and neoxanthin and total carotenoids. Additional strong positive correlations were observed between GSSG and zeaxanthin, GSSG and A+Z, and between A+Z and zeaxanthin.

At day 3, strong correlations were observed in both species, with increased connectivity compared to the 0th day of experiment. In *M. alba*, strong positive correlations were observed between cysteine and total GSH, and between GSSG and cystine. Strong positive correlations were also present within the photosynthetic pigments, including zeaxanthin and total carotenoids, and A+Z with total carotenoids, as well as between A+Z and zeaxanthin. No strong negative correlations were prominently detected across major metabolite groups. In *M. indica*, multiple strong correlations were identified. Strong negative correlations were observed between total carotenoids and TPC, and between chl b and TPC. Additional strong negative correlations were present between pigments and Cu. Strong positive correlations were detected between GSSG and zeaxanthin, GSSG and A+Z, and between A+Z and zeaxanthin. Further strong correlations were observed among TPC, pigments and thiols.

At day 7, strong correlations remained evident in both species. In *M. alba*, strong positive correlations were detected within cysteine and total GSH, and GSSG and cystine. Strong positive correlations were also observed within the pigments, including zeaxanthin and total carotenoids, and A+Z with both zeaxanthin and total carotenoids. Strong negative correlations were not observed. In *M. indica*, strong negative correlations were observed between total carotenoids and TPC, and between chl b and TPC. Strong negative correlations were also present between pigments and Cu content. Strong positive correlations were detected between GSSG and zeaxanthin, GSSG and A+Z and between total A+Z and zeaxanthin. Additional strong correlations were observed among pigments, TPC, and thiol-related metabolites.

At day 21, strong correlations among different determined metabolites persisted in both species. In *M. alba*, strong positive correlations were observed between cysteine and total GSH, and between GSSG and cystine. Strong positive correlations were also observed within the pigments, including zeaxanthin and total carotenoids and A+Z with both zeaxanthin and total carotenoids. Only a limited number of strong negative correlations were observed. In *M. indica*, numerous strong correlations were identified across metabolic groups. Strong negative correlations were observed between photosynthetic pigments and TPC, and between Cu and multiple metabolites. Strong positive correlations were detected between GSSG and zeaxanthin, GSSG and A+Z, and among oxidized redox-related parameters, including cystine and DHA. Strong correlations were also observed within pigment groups and between pigments and TPC.

## 3. Discussion

### 3.1. Visible Symptoms and Shoot Growth Under Cu Stress

The present study demonstrated species- and variety- specific differences in the response of mulberry to Cu-induced stress. CuSO_4_ treatment caused visible chlorosis and reduced shoot growth in all tested varieties, but the magnitude of these responses differed considerably. The most pronounced chlorosis and strongest inhibition of shoot growth were observed in ‘Kokusou’, whereas ‘Florio’ and ‘Giazzola’ showed comparatively minor visible symptoms and maintained the highest relative shoot growth.

Chlorosis is a well-established symptom of excessive Cu exposure [14], and similar responses have been reported in *M. alba* [50] and *M. nigra* [51]. Excess Cu can damage chloroplast ultrastructure, including thylakoid membranes and grana, while its redox activity promotes ROS formation and oxidative damage to membrane lipids and proteins [16,52,53]. The visible symptoms observed in the present study are therefore consistent with established effects of Cu toxicity.

The inhibition of shoot growth further demonstrates that Cu exposure affected plant development in a variety-dependent manner. Excess Cu can reduce nitrate uptake and protein synthesis, inhibit cell division and cause oxidative damage to proteins, lipids, DNA and RNA, thereby limiting shoot elongation and biomass accumulation [54,55,56]. ROS-induced impairment of chloroplast electron transport and photosynthetic enzymes may additionally reduce carbon fixation and ATP/NADPH production, further restricting growth [53,57]. Similar Cu-induced growth inhibition has been reported in mulberry [58], as well as in wheat and rice [59,60]. In the present study, however, the extent of growth inhibition varied substantially among varieties. The relatively small reduction in shoot growth in ‘Florio’ and ‘Giazzola’, together with their limited chlorosis, indicates a greater capacity to maintain growth under the applied Cu exposure, whereas the strong growth inhibition and severe chlorosis in ‘Kokusou’ indicate greater sensitivity.

The relationship between visible symptoms and growth is particularly relevant when considered together with the biochemical responses. Varieties with shoot growth comparable to controls generally showed stronger antioxidant responses and better preservation of PSII efficiency and photosynthetic pigments’ values. This indicates that growth inhibition was not an isolated response but was associated with broader physiological disturbance. Conversely, the varieties with the strongest visible symptoms showed progressive oxidation of redox pools and greater degradation of photosynthetic pigments. Thus, the morphological and growth responses provide an integrative phenotype reflecting the cumulative effects of Cu-induced stress.

### 3.2. Cu Accumulation in Leaves

Under control conditions, leaf Cu contents were comparable with previously reported values for *M. alba* and *M. indica* [61,62], while differences among species were consistent with previously documented genetic variation in Cu accumulation between *M. alba* and *M. indica* [63]. Such variation may result from differences in nutrient uptake, translocation and homeostasis [16]. The relatively stable Cu contents observed in control plants throughout the experiment are also consistent with tight regulation of Cu homeostasis in fully developed leaves [2].

CuSO_4_ treatment increased leaf Cu content in all varieties, in agreement with previous observations in spinach, parsley and *Corchorus capsularis* exposed to Cu [64,65]. Cu uptake and distribution depend on Cu availability in the rhizosphere and on transport systems that regulate uptake, xylem loading and distribution to aerial tissues [66,67]. Under elevated external Cu concentrations, increased transport to shoots can consequently result in higher Cu accumulation in leaves [68,69].

A notable finding of the present study was that Cu accumulation was more pronounced and more persistent in *M. alba* than in *M. indica*. The leaf Cu concentrations in the *M. alba* varieties generally peaked on day 7 and remained higher at day 21 than those measured in the *M. indica* varieties. Importantly, the greater Cu accumulation in *M. alba* was not accompanied by greater physiological damage. Instead, *M. alba* varieties generally maintained better shoot growth, redox regulation and photosynthetic performance. This indicates that leaf Cu concentration alone does not necessarily reflect Cu sensitivity. One possible explanation is that the *M. alba* varieties possess a greater capacity to detoxify or compartmentalize absorbed Cu. Excess Cu can be bound by phytochelatins, metallothioneins or GSH and subsequently sequestered in vacuoles or cell walls, thereby reducing the concentration of free Cu ions in metabolically active compartments [70,71]. The higher and more persistent leaf Cu accumulation in *M. alba* may therefore reflect a greater capacity to retain Cu in relatively less toxic forms rather than inefficient regulation. In contrast, the lower leaf Cu concentrations in *M. indica* could reflect more restricted uptake or translocation to leaves [2,66]. However, these mechanisms were not directly measured in the present experiment and should therefore be regarded as possible explanations rather than demonstrated mechanisms. The temporal pattern of Cu accumulation, with maximum values generally occurring at day 7 followed by lower concentrations at day 21, may indicate an initial phase of rapid uptake and translocation followed by stronger regulation of Cu transport and intracellular sequestration [16,56]. The persistence of higher Cu concentrations in *M. alba* at day 21, together with their comparatively better physiological performance, is consistent with the possibility of more effective Cu sequestration. Nevertheless, because only one Cu concentration was tested and root Cu content was not measured, the present results cannot establish a dose–response relationship or distinguish differences in uptake, root retention, root-to-shoot translocation and leaf sequestration.

Root Cu accumulation is particularly important for interpreting overall plant Cu tolerance because roots may retain substantial amounts of absorbed Cu before its translocation to shoots [72]. Root measurements were not included in the present experiment because repeated destructive root sampling would have required independent plants at each sampling time and root disturbance itself can induce defence-related responses [73]. Thus, the absence of root Cu data represents an important limitation. Future experiments should combine several Cu concentrations with independent root and shoot sampling to determine whether the contrasting leaf Cu concentrations arise from differences in uptake, root retention or translocation.

### 3.3. Redox Regulation—ASC–GSH System and Low-Molecular-Weight Thiols

Under control conditions, total ASC, cysteine and GSH contents and their oxidized fractions remained relatively stable during the experiment, although basal differences among varieties were observed. Such variation is consistent with the genotype-dependent regulation of antioxidant and thiol metabolism reported in mulberry and other plants. Total ASC, cysteine and total GSH contents were also consistent with previous studies [74,75,76,77]. The relatively low DHA ratio under control conditions indicates that the ascorbate pool was maintained predominantly in its reduced state [78,79,80]. Similarly, the low cystine and GSSG ratios indicate that the glutathione and thiol pools were maintained predominantly in their reduced state, consistent with the generally high reduction state of glutathione in unstressed leaves [77,80,81].

Cu exposure induced distinct temporal patterns in the ASC pool among the tested varieties. In ‘Florio’ and ‘Giazzola’, total ASC increased strongly during the first 7 days and subsequently decreased, while remaining above the initial level. The DHA ratio increased during the early phase and then declined, indicating an initial increase in ASC oxidation followed by partial restoration of the reduced ASC pool. A similar increase in ASC together with an elevated DHA pool was previously reported in *M. alba* ‘Kanva 2’ exposed to Cu [50]. The subsequent stabilization of the ASC pool in ‘Florio’ and ‘Giazzola’ is consistent with active regeneration of ASC through the ASC–GSH cycle, which is important for maintaining redox homeostasis under oxidative stress [79,80,82]. In ‘Budapest’, ASC initially decreased and subsequently recovered, whereas the DHA ratio remained relatively high. This pattern suggests that ASC oxidation persisted for longer and that restoration of the reduced ASC pool was slower than in ‘Florio’ and ‘Giazzola’ [80,82]. In both *M. indica* varieties, the continuous decrease in total ASC accompanied by an increasing DHA ratio indicates progressive oxidation of the ASC pool [80,83,84,85]. Thus, the temporal behaviour of ASC suggests a greater capacity of the *M. alba* varieties, particularly ‘Florio’ and ‘Giazzola’, to maintain ascorbate redox balance during prolonged Cu exposure.

The low-molecular-weight thiols showed a related but distinct response. In ‘Florio’ and ‘Giazzola’, cysteine and total GSH increased markedly during the first 7 days of Cu exposure, whereas cystine and GSSG ratios increased during the early phase and subsequently declined. The increase in cysteine is consistent with its role as a precursor for GSH biosynthesis, while the increase in GSH indicates activation of thiol metabolism during Cu exposure [76,77,86,87]. The simultaneous increase in cystine and GSSG indicates enhanced oxidation of thiol pools during the early phase of Cu stress [76,88]. Their subsequent decline, together with the maintenance of elevated cysteine and GSH contents, is consistent with partial restoration of the reduced thiol pool. A comparable response of GSH pools to metal stress has been reported in plants exposed to Cd [89]. The early increase in reduced thiols therefore suggests that ‘Florio’ and ‘Giazzola’ were able to increase thiol availability while subsequently restoring their redox balance. ‘Budapest’ showed a different temporal pattern. Cysteine increased during the first 7 days and then declined, while total GSH increased during the first 3 days and subsequently decreased to a level slightly below the initial value. In parallel, cystine and GSSG ratios increased and remained relatively high at the later sampling dates. The initial increase in cysteine and GSH is consistent with enhanced sulfur assimilation and GSH biosynthesis, whereas their subsequent decline may reflect continued demand for GSH in redox regulation and metal detoxification [86,87,90,91]. GSH is involved in heavy-metal tolerance through antioxidant and redox functions and can also provide thiol groups for phytochelatin formation [76,87,90,91,92]. The persistence of relatively high oxidized thiol fractions in ‘Budapest’ therefore indicates that its thiol system remained under greater oxidative demand during prolonged Cu exposure than that of ‘Florio’ and ‘Giazzola’. The strongest disruption of thiol redox status occurred in the *M. indica* varieties. In ‘Kokusou’ and ‘Shin-Tso’, cysteine and GSH showed an overall decline during the experiment, accompanied by increasing cystine and GSSG ratios. The particularly high oxidized thiol fractions at the later sampling dates indicate a pronounced shift in the thiol pools towards their oxidized forms. Similar alterations in GSH metabolism have been reported under toxic-metal stress, where changes in GSH/GSSG balance are associated with oxidative stress and the demand for redox buffering [88,89,91,93,94]. The concurrent decline in reduced ASC and GSH and increase in their oxidized fractions therefore indicates a progressive loss of redox homeostasis in the *M. indica* varieties during prolonged Cu exposure.

Taken together, the ASC and low-molecular-weight thiol responses indicate that the capacity to activate antioxidant metabolism and subsequently restore the reduced state of redox pools was an important component of the variety-specific response to Cu stress. ‘Florio’ and ‘Giazzola’ combined an early increase in ASC, cysteine and GSH with subsequent decreases in DHA, cystine and GSSG, indicating more effective recovery of redox balance. ‘Budapest’ showed a similar response but maintained higher oxidized thiol fractions for longer, whereas ‘Kokusou’ and ‘Shin-Tso’ showed progressive oxidation and depletion of both ASC and thiol pools. These coordinated responses support a role for ASC–GSH metabolism and thiol-dependent detoxification in the differential response of the tested mulberry varieties to Cu treatment [76,79,82,87,90]. However, because enzyme activities, phytochelatins and metal-binding proteins were not determined, the underlying mechanisms remain inferred from metabolite patterns. Direct measurements of ASC–GSH cycle enzymes, sulfur-assimilation enzymes and thiol-based metal-chelation pathways would therefore be required to confirm these mechanisms [86,87,90,92].

### 3.4. Phenolic Compounds

TPC was relatively stable in fully developed leaves under control conditions, while differences among varieties were consistent with previously reported variation in mulberry phenolic composition [95,96]. Such differences may reflect genetic regulation of phenolic metabolism and the influence of developmental and environmental conditions [96,97]. Under Cu exposure, however, TPC showed clear variety-specific temporal responses.

In ‘Budapest’ and ‘Giazzola’, TPC increased during the first 7 days and subsequently declined slightly but remained above the initial level. A comparable early increase in phenolic compounds has been observed under heavy-metal stress in the common bean [98]. Heavy-metal exposure can stimulate phenylpropanoid metabolism, increasing phenolic biosynthesis as part of the antioxidant response [92]. The subsequent decline may reflect metabolic acclimation, redistribution or utilization of phenolic compounds during prolonged stress [99]. Thus, the maintenance of elevated TPC in these varieties indicates that phenolic metabolism remained active during prolonged Cu exposure. ‘Florio’ showed the strongest and most sustained increase in TPC, with the highest values reached during the later stages of the experiment. A similar temporal pattern was observed in *M. nigra* stem-node cultures exposed to CuSO_4_ [51]. Phenolic compounds can contribute to stress protection by scavenging ROS and interacting with metal ions, while flavonoids can additionally contribute to metal chelation [26,28]. The sustained increase in TPC in ‘Florio’, together with its strong ASC–GSH system response and preservation of photosynthetic pigments, suggests that phenolic metabolism formed part of a broader and coordinated antioxidant response rather than acting independently.

The *M. indica* varieties displayed a less sustained phenolic response. TPC increased during the first 7 days but subsequently declined, more strongly, and in ‘Kokusou’ the day-21 value was lower than the initial value. A similar decline in phenolic content has been reported under Cu stress in tartary buckwheat [99]. Such a response may indicate weaker activation or reduced capacity to maintain the phenylpropanoid pathway during prolonged Cu exposure [26]. Depletion of antioxidant reserves or inhibition of phenolic biosynthesis under prolonged metal stress may also contribute to this decline.

The temporal differences in TPC therefore correspond with the broader physiological responses observed in the present study. The sustained phenolic response in *M. alba*, particularly in ‘Florio’, occurred together with stronger redox regulation, better maintenance of photosynthetic pigments and higher shoot growth, whereas the less sustained response in *M. indica* coincided with greater redox oxidation and pigment loss. These relationships support a role for phenolic metabolism in Cu stress tolerance, although they do not demonstrate a direct causal relationship. TPC is an aggregate measure and does not identify the individual compounds responsible for the response. Different phenolics can have different antioxidant and metal-chelating capacities [26,100]. Future studies should therefore determine individual phenolic profiles and the activity or expression of key enzymes of the phenylpropanoid pathway, including PAL, CHS, C4H and 4CL [26,101]. Such analyses would help determine whether the stronger TPC response observed in the more tolerant varieties reflects increased synthesis of specific protective phenolics.

### 3.5. Photosynthetic Pigments, Photoprotection and PSII Efficiency

Cu exposure strongly affected the photosynthetic apparatus, but the magnitude and temporal development of these effects differed among varieties. Under control conditions, chl and carotenoid contents were comparable with previously reported values [102], and differences among varieties likely reflected genetic variation in pigment metabolism and organization of the photosynthetic apparatus [103,104]. The stability of chl content in control plants is consistent with the tight regulation of pigment levels in fully developed leaves [105].

After CuSO_4_ treatment, chl a and chl b decreased progressively in all varieties. Similar Cu-induced chl losses have been reported in *Aegilops tauschii* [106]. Excess Cu can promote ROS formation, damage chloroplast membranes and interfere with chl biosynthesis and photosynthetic electron transport [84,107]. The decrease was less pronounced in *M. alba*, particularly in ‘Giazzola’ and ‘Florio’, whereas both *M. indica* varieties showed substantially greater pigment losses. The stronger preservation of chl in *M. alba* is consistent with more effective antioxidant protection and better maintenance of chloroplast function [83,84]. The decline in the chl a/b ratio, particularly in *M. indica*, provides additional evidence of disruption of the photosynthetic apparatus [108].

Total carotenoids showed a different temporal response. ‘Florio’ and ‘Giazzola’ increased carotenoid content during the first 7 days and retained comparatively high levels at day 21, whereas ‘Kokusou’ and ‘Shin-Tso’ showed a continuous decline. ‘Budapest’ displayed an intermediate response. Because carotenoids contribute to ROS protection and dissipation of excess excitation energy [109,110], the early increase and subsequent maintenance of carotenoids in ‘Florio’ and ‘Giazzola’ are consistent with active photoprotection under Cu stress. The contrasting decline in *M. indica* suggests a reduced capacity to maintain photoprotective pigment homeostasis during prolonged exposure [83,84].

The individual xanthophyll responses further demonstrated dynamic and variety-specific photoprotection. Under control conditions, neoxanthin and lutein differed among varieties, whereas the main xanthophyll-cycle pigments showed less pronounced variation. These pigments are components of the photosynthetic antenna and photoprotective system, with the xanthophyll cycle providing a dynamic mechanism for the dissipation of excess excitation energy [111,112,113]. Under Cu exposure, neoxanthin decreased progressively in ‘Budapest’ and in both *M. indica* varieties, whereas ‘Florio’ and ‘Giazzola’ showed early increases followed by partial declines while retaining higher levels than at the beginning of the experiment. Lutein similarly increased during the early phase in ‘Florio’ and ‘Giazzola’ and remained elevated at day 21, whereas it declined continuously in both *M. indica* varieties. Lutein is an important component of photoprotection and contributes to the regulation of excess excitation energy [113]. The maintenance of elevated lutein and neoxanthin in ‘Florio’ and ‘Giazzola’ therefore supports the interpretation that these varieties maintained a stronger photoprotective response.

The xanthophyll cycle provided further evidence of active adjustment to Cu-induced stress. In ‘Florio’ and ‘Giazzola’, the early decrease in the V/A+Z ratio was accompanied by an increase in A+Z, followed by partial recovery of the pigment balance. This pattern is consistent with activation of xanthophyll-cycle-dependent non-photochemical energy dissipation [84,112,113]. ‘Budapest’ also showed early activation but less complete restoration by day 21. In contrast, the *M. indica* varieties showed a more persistent shift toward de-epoxidized xanthophylls, together with continuous losses of violaxanthin. This combination suggests sustained photoprotective demand accompanied by a reduced capacity to restore pigment homeostasis [83,84,113,114]. The contrasting temporal responses therefore indicate that the more tolerant *M. alba* varieties were able to maintain a more dynamic photoprotective adjustment, whereas the *M. indica* varieties showed progressive depletion of several pigment pools under prolonged Cu exposure.

The functional relevance of these pigment responses was supported by Fv/Fm measurements. CuSO_4_ treatment significantly reduced Fv/Fm in all varieties, indicating impaired PSII efficiency. Similar reductions in Fv/Fm under Cu-induced oxidative stress have been associated with ROS-mediated damage to PSII proteins, disruption of electron transport and impaired charge separation [83,84,114]. The magnitude of the decline differed among varieties: ‘Giazzola’ retained the highest Fv/Fm under Cu exposure, whereas ‘Kokusou’ showed the lowest value. The stronger decrease in both *M. indica* varieties corresponded with their greater losses of chl and carotenoids and their less sustained redox and photoprotective responses.

Thus, the photosynthetic measurements provide a functional link between biochemical protection and plant performance. Varieties that maintained stronger ASC–GSH cycle response and phenolic responses also generally preserved more chl and carotenoids, showed more dynamic xanthophyll-cycle regulation and maintained higher Fv/Fm. Effective maintenance of photosynthetic efficiency under metal stress depends on the preservation of the photosynthetic apparatus, antioxidant protection and regulation of excess excitation energy [84,115,116]. The coordinated pigment and Fv/Fm responses therefore support the interpretation that effective redox regulation and photoprotection contributed to the maintenance of photosynthetic function under Cu exposure.

### 3.6. Integrated Biochemical Responses and Metabolic Relationships

The individual biochemical responses demonstrate that Cu stress affected interconnected physiological processes rather than a single metabolic pathway. The two-way ANOVA showed significant effects of variety, CuSO_4_ treatment, sampling time and their interactions on most measured biochemical parameters, confirming that the response was both variety-dependent and dynamic. The observed changes in ASC, cysteine, GSH, phenolics, photosynthetic pigments and xanthophyll pigments contents therefore represent coordinated but temporally distinct components of the response to Cu exposure [2,80,84].

The hierarchical clustering analysis provided an integrated view of these responses. The major clusters separated the *M. indica* and *M. alba* varieties, with control and Cu-treated samples occurring within their respective species clusters. This indicates that intrinsic species-related physiological and biochemical characteristics contributed strongly to the overall separation of the samples. Within *M. indica*, control and Cu-treated plants were more clearly separated, whereas the *M. alba* samples showed greater overlap and more variety-dependent clustering. These patterns are consistent with stronger and more uniform metabolic disturbance in *M. indica* and greater heterogeneity and flexibility in the response of *M. alba* varieties.

The Pearson correlation analysis further showed that the relationships among biochemical parameters changed over time. In *M. alba*, strong correlations remained largely within functional groups, particularly thiol metabolism and the xanthophyll cycle. Associations between cysteine and GSH and among zeaxanthin, A+Z and total carotenoids indicate coordinated regulation of antioxidant and photoprotective processes. Such organization may allow individual metabolic systems to respond to stress while retaining a degree of functional independence [84,112]. In contrast, *M. indica* exhibited a denser network of cross-pathway correlations. Negative relationships between photosynthetic pigments and TPC or Cu content, together with positive relationships between oxidized redox markers and xanthophyll-cycle components, indicate stronger coupling between oxidative stress, secondary metabolism and photosynthetic responses. The persistence of these associations through the later stages of the experiment is consistent with prolonged metabolic disturbance rather than stabilization of individual pathways [80,84].

The correlation patterns should nevertheless be interpreted as associations rather than evidence of direct causality. Their biological value lies in showing that the different measurements did not change independently. Instead, the stronger tolerance-associated phenotype of *M. alba* was accompanied by a more structured response in which redox regulation and photoprotection remained comparatively coordinated, whereas the *M. indica* varieties exhibited stronger cross-pathway disruption. Together with the clustering results, these analyses support the interpretation that Cu tolerance in mulberry involves coordinated regulation across antioxidant metabolism, secondary metabolism and photosynthetic protection rather than dependence on a single biochemical marker.

### 3.7. Variety-Specific Cu Tolerance and Overall Interpretation

Considering the complete set of morphological, physiological and biochemical observations, the tested varieties showed distinct patterns of response to the applied Cu-treatment. ‘Florio’ and ‘Giazzola’ consistently combined relatively low visible toxicity, better maintenance of shoot growth, strong early activation and subsequent stabilization of ASC–GSH cycle and thiol metabolism, sustained phenolic responses, preservation of chl and carotenoids, dynamic xanthophyll-cycle adjustment and comparatively high Fv/Fm. These responses collectively indicate a greater capacity to maintain physiological homeostasis under the Cu conditions applied in the present experiment [50,58,83,84].’Budapest’ displayed an intermediate response. It activated antioxidant, phenolic and photoprotective mechanisms, but several of these responses were less sustained during prolonged exposure. The relatively high oxidized ASC and thiol fractions at later sampling dates, together with the less sustained pigment response, indicate that its capacity to maintain redox and photosynthetic homeostasis was lower than that observed in ‘Florio’ and ‘Giazzola’ [80,83,84]. The two *M. indica* varieties exhibited the strongest overall evidence of physiological disturbance. Their responses included greater chlorosis and shoot-growth inhibition, progressive depletion and oxidation of ASC and thiol pools, less sustained phenolic accumulation, stronger losses of chl and carotenoids, persistent changes in xanthophyll-cycle pigments and greater reductions in Fv/Fm. ‘Kokusou’ generally exhibited the strongest changes, particularly in the oxidized redox fractions and Fv/Fm, corresponding with its pronounced visible symptoms and growth inhibition [14,54,83,84].

An important finding was that the varieties showing better physiological performance also accumulated more Cu in leaves. This indicates that higher tissue Cu concentration cannot be interpreted as a direct indicator of greater Cu sensitivity. Instead, greater accumulation may be compatible with tolerance when Cu is effectively chelated or compartmentalized, thereby limiting the concentration of free Cu in sensitive cellular compartments [56,70,71]. The present data are therefore consistent with the possibility that *M. alba* varieties, particularly ‘Florio’ and ‘Giazzola’, possess more effective mechanisms for maintaining cellular homeostasis in the presence of elevated leaf Cu. However, because Cu localization, transporter activity, metallothioneins and phytochelatins were not measured, this mechanism remains to be experimentally confirmed.

The integrated findings also place the present results in the context of previous mulberry research. Previous studies have mainly examined Cu or heavy-metal responses in individual *M. alba* genotypes or other *Morus* species [48,50,51,58], whereas the present study directly compared three *M. alba* and two *M. indica* varieties under the same Cu exposure while simultaneously assessing growth, leaf Cu accumulation, redox metabolism, phenolic compounds, photosynthetic pigments, xanthophyll-cycle pigments and PSII efficiency. The consistent differences observed across these independent levels of organization provide evidence that the contrasting responses of the tested varieties were not restricted to a single metabolic pathway.

Overall, the results support a model in which greater Cu tolerance is associated with maintenance of growth, Cu homeostasis, antioxidant redox balance, phenolic metabolism and photosynthetic protection. In the more tolerant *M. alba* varieties, early activation of ASC–GSH cycle, phenolic metabolism and photoprotective responses was followed by partial stabilization of these responses, allowing photosynthetic function and shoot growth to be maintained. In contrast, the *M. indica* varieties showed progressive oxidation of redox pools, greater pigment losses and stronger PSII impairment, coinciding with greater growth inhibition and visible toxicity. The results therefore support the conclusion that ‘Florio’ and ‘Giazzola’ have greater potential capacity to tolerate the applied Cu exposure than the other varieties investigated [80,83,84].

This conclusion should, however, be considered within the limitations of the experimental design. Only one Cu concentration was tested, and Cu accumulation was assessed in leaves but not roots. The present experiment therefore provides evidence of relative potential tolerance under a defined Cu exposure rather than a complete dose–response characterization. Future studies should include several Cu concentrations, independent root and shoot sampling, Cu localization and measurements of Cu transporters, metallothioneins, phytochelatins and key antioxidant enzymes. Such experiments would help distinguish differences in Cu uptake, root retention, translocation and intracellular detoxification and would provide a more direct mechanistic basis for the variety-specific responses observed in the present study [66,70,72].

## 4. Materials and Methods

### 4.1. Chemicals

CuSO_4_, HPLC-grade acetonitrile, HPLC-grade methanol, HPLC-grade toluene and sodium carbonate (Na_2_CO_3_) were obtained from Honeywell (Charlotte, NC, USA). Monobromobimane was bought from Life Technologies (Eugene, OR, USA). Folin–Ciocalteu’s phenol reagent was purchased from Merck (Darmstadt, Germany). HPLC-grade acetic acid was supplied by Molar Chemicals KFT (Halásztelek, Hungary). 2-Amino-2-(hydroxymethyl)-1,3-propanediol (TRIS buffer), 2-(cyclohexylamino)ethanesulfonic acid (CHES buffer), dithiothreitol (DTT), HPLC-grade acetone, HPLC-grade ethyl acetate, HPLC-grade formic acid, HPLC-grade isopropanol, hydrochloric acid (HCl), hydrogen peroxide (H_2_O_2_), metaphosphoric acid, methansulfonic acid, N-ethylmaleimide (NEM), nitric acid (HNO_3_), ortophosphoric acid, polyvinylpyrrolidone (PVP), sodium dihydrogen phosphate (NaH_2_PO_4_) and standard compounds ascorbic acid, gallic acid, *L*-cysteine, *L*-glutathione and xanthophyll were obtained from Sigma-Aldrich (Hamburg, Germany). The ultra-pure water (resistance above 18 MΩ cm) used was obtained from a Milli-Q water purification system.

### 4.2. Plant Material

A pot experiment on tolerance to CuSO_4_ in mulberries of different varieties was carried out in the greenhouse of the Faculty of Agriculture and Life Sciences, University of Maribor (Slovenia; 46°30′17.4″ N, 15°37′34.6″ E), during May 2024 and June 2024. Three varieties of the *M. alba*—‘Budapest’, ‘Florio’ and ‘Giazzola’—and two varieties of *M. indica*—‘Kokusou’ and ‘Shin-Tso’—were included in the experiment. All container-grown two-year old trees were vegetatively propagated from green cuttings. The trees from which the material for the preparation of green cuttings originated were planted in the mulberry collection of the University of Maribor (φ: 46°50′88.33″; γ: 15°62′20.73″). More detailed information about the varieties included in the present study is presented in Table 3. Three months before the beginning of the experiment, plants were transplanted into plastic pots with a diameter of 28 cm using 12 L Klassman T3 substrate each (Klasmann-Deilmann GmbH, Geeste, Germany). The substrate consisted of white sphagnum peat H3–H5 with added clay and NPK fertilizer, intended for general horticultural pot cultivation (pH in H_2_O: 5.5–6.0; electrical conductivity: ~1.0 mS cm^−1^; dry bulk density: ~130–150 kg m^−3^; N: 140 mg L^−1^; P_2_O_5_: 120 mg L^−1^; K_2_O: 180 mg L^−1^; Mg: 50 mg L^−1^; Ca: 150 mg L^−1^ as carbonate; salt content: 1.0 g L^−1^ KCl; grain size: 0–25 mm). The substrate with a slightly acidic pH was chosen because the free Cu^2+^ species predominates at acidic pH. In a substrate with a pH of 6.0, 98% of Cu is available as Cu^2+^ [117]. Six two-year old trees of each variety were included in the experiment—three were cultivated under control conditions and three were exposed to CuSO_4_ treatment. On day 0 (the beginning of the experiment), plants cultivated under control conditions were watered with 2 L of tap water obtained from the local water supply (supplier Mariborski vodovod, Maribor, Slovenia). Plants treated with CuSO_4_ were watered on day 0 of the experiment with 2 L of a CuSO_4_ solution in tap water, in which the appropriate amount of CuSO_4_ was added, to enrich the substrate with a final concentration of 150 mg Cu^2+^ kg^−1^ substrate. The calculated concentration of dissolved CuSO_4_ was calculated based on the dry mass of 12 L of substrate (the amount of substrate in which the plants were planted), so that these 12 L of substrate were ultimately enriched with 150 mg Cu^2+^ kg^−1^ of substrate. As the Cu source, CuSO_4_ was used, because most agricultural areas are contaminated with this form of Cu [6], while the substrate was enriched to a concentration of 150 mg Cu^2+^ kg^−1^ of substrate, because concentrations above 100 mg Cu^2+^ kg^−1^ are toxic to plants and are also exceeded in many vineyard areas. In addition, exceeded contents of between 100 and 150 mg Cu kg^−1^ are still permissible [15,118,119]. The experiment was conducted for 21 days. The plants were kept in a greenhouse throughout the experiment, as well as for the entire three months before the beginning of the experiment, with a mean air temperature of 26 ± 1 °C, a relative humidity of 70 ± 2%, and with controlled drip irrigation. All plants were watered with 1 L of tap water every alternate day throughout the experiment. On day 0 of the experiment, before application of the CuSO_4_, all plants were checked for visible morphological characteristics, their shoot lengths were measured, and leaves were sampled for the purpose of analyzing selected metabolites. The day 0 leaf samples for biochemical analyses were collected from all plants of each variety before treatment application, and the biochemical measurements obtained from these samples were averaged to establish the initial biochemical status of each variety. These mean day 0 values were subsequently used as the common baseline for the control and CuSO_4_-treated groups. On the last (21st) day of the experiment, all plants were checked again for visible morphological characteristics, shoot lengths were measured, Fv/Fm in leaves was measured with fluoropen, and leaves were sampled again for the purpose of analyzing selected metabolites. In addition to these two leaf samplings for the purpose of metabolite analysis, leaves were also sampled on the 3rd and 7th day of the experiment. Leaf samplings were carried out according to Šelih et al. [120]. Five to seven sun-exposed, fully developed leaves (the fifth to seventh leaves below the apex) were randomly collected from each sampled plant on a current-year shoot, avoiding shoots sampled on the previous sampling date. Samples were immediately stored on dry ice and transferred to a freezer at −80 °C, subsequently freeze-dried (Lyophilizer Alpha, Christ, Osterode am Harz, Germany) and grounded (TissueLyser II, Qiagen, Hilden, Germany). The grounded samples were then stored in air-tight vials at −20 °C prior to biochemical analyses.

### 4.3. Symptom Monitoring

Morphological changes were evaluated on day 0 of the experiment. The assessment of the visual quality of plants was done at the beginning of the experiment (day 0) and on the 21st day of the experiment based on observable characteristics—variations in leaf colour, and the appearance of chlorosis, necrosis, deformations and asymmetries. Leaf morphological differences were assessed on sun-exposed fifth to seventh leaves below the apex, which were fully developed on the day of morphological assessment. Five leaves were observed on each observed plant.

### 4.4. Relative Growth Rate of Shoots

To analyze the effect of Cu on the growth of shoots, the relative growth rate of shoots (in %) was calculated according to Vršič et al. [118]. Relative growth rate of shoots is defined as the ratio between the length of shoots (in cm) of plants developing in control pots (La) and the length of plant shoots developing in pots (Lb) with substrate enriched with 150 mg Cu^2+^ kg^−1^. The relative growth rate was calculated as (Lb/La) × 100. Values below 100% indicate growth inhibition, and values above 100% indicate growth acceleration. Shoot lengths were measured with a tape measure as the distance from the point of growth of the shoot to the end point of the shoot. Measurements were performed on the 0th and 21st day of the experiment on the same current-year shoot. Three current-year shoots were observed on each observed plant.

### 4.5. Determination of Cu Content

The Cu content in the samples was determined according to the standard method EN 14082:2003 [121]. In total, 250 mg of lyophilized sample was weighed into dry digestion vessels, and 12 mL of 70% HNO_3_, 2 mL of 30% H_2_O_2_ and 20 mL of ultra-pure water were added. The vessels were placed into the microwave digestion system NMDS-200 (Labnics Equipment Ltd., Glasgow, UK). After cooling, the obtained solutions were transferred into a 50 mL volumetric flask and diluted with ultra-pure water to a total volume of 50 mL. Solutions were filtered before they were transferred to test tubes for analysis by Atomic Absorption Spectrometer Varian AA 240FS (Agilent Technologies, Santa Clara, CA, USA) and by reading of its absorbance at 324.8 nm. The standard curve covered the concentration range from 10 µg/L to 1000 µg/L of Cu. The limit of quantification was above 20.00 mg kg DW^−1^. Results are presented as mg Cu kg DW^−1^. All sample preparations and all measurements were performed in duplicate.

### 4.6. Extraction and Determination of ASC and Its Reduced Form

The content of total ASC and the content of reduced ASC were determined according to Tausz et al. [122]. The standard curve covered the concentration range from 416.3 nmol/mL to 2489 nmol/mL of ASC. The standards were treated according to the same procedure as the samples in which the content of the reduced form of ASC was determined. Each sample was extracted and prepared for analysis of both forms in duplicate. The analysis was done using an isocratic reversed-phase chromatography method. The HPLC analyses were performed using an HPLC-PDA chromatography system (HPLC Waters Alliance 2695 System, coupled with a 2996 photodiode array detector). The separation was performed on a Phenomenex Synergy 4 μm Hydro-RP 80 A column (150 × 4.60 mm). The mobile phase was 32.5 mM NaH_2_PO_4_ (pH 2.2). ASC was detected at a wavelength of 250 nm. The run time was 20 min with a flow rate of 0.5 mL/min. The results are expressed in nmol/g DW. Each sample vial was analyzed twice.

### 4.7. Extraction and Determination of Low Molecular Thiols and Their Oxidized Form

The content of cysteine, total GSH and the content of cystine and GSSG were determined according to Kranner and Grill [123]. The cysteine standard curve covered the range from 1.42 nmol/mL to 19.88 nmol/mL and the GSH standard curve covered the range from 2.79 nmol/mL to 39.18 nmol/mL. The standards were prepared according to the same procedure as the samples in which the cysteine and total GSH contents were determined. Each sample was extracted and prepared for analysis of both forms in duplicate. The HPLC analyses were performed using an HPLC-FLD chromatography system (HPLC Waters Alliance 2695 System, coupled with a 2475 Fluorescence detector; Waters, Milford, Massachusetts, USA). Waters Grace Spherisorb ODS-2.5 μm, 250 × 4.6 mm column was used. The run time was 40 min with a flow rate of 1 mL/min. Mobile phase A contained double-distilled water with 5% methanol and 0.25% acetic acid, pH 3.9, while mobile phase B contained 90% methanol with 0.22% acetic acid in water, pH 3.9. The gradient consisted of 20 min of 90% solvent A and 10% solvent B, 20–30 min of 85% solvent A and 15% solvent B, 30–35 min of 5% solvent A and 95% solvent B, and 35 min of 90% solvent A and 10% solvent B. The results are expressed in nmol/g DW. Each sample vial was analyzed twice.

### 4.8. Extraction of Phenolic Compounds and Determination of Total Phenolic Content

Phenolic compounds were extracted according to Senekovič et al. [51]. TPC was determined spectrophotometrically using the Folin–Ciocalteu method [124]. The absorbance of the standards and the samples were measured with a UV-ViS spectrophotometer (Varian Cary 50 Bio, Varian, Palo Alto, CA, USA) at 765 nm against a blank. Gallic acid-containing standard solutions were prepared in 70% methanol (acidified in 3% formic acid) at concentrations ranging from 0.052 to 0.280 mg mL^−1^ to generate the calibration curve. TPC was expressed as milligrams of gallic acid equivalents per gram dry weight of plant material (mg GA g^−1^ DW). All samples were extracted and analyzed in duplicate.

### 4.9. Extraction and Determination of Photosynthetic Pigments

Photosynthetic pigments were extracted according to Pfeifhofer [125]. The contents of chl a, chl b and total carotenoids were determined spectrophotometrically (Varian Cary 50 Bio spectrophotometer, Varian, Palo Alto, CA, USA) according to Lichtenthaler and Buschmann [126]. The absorbance of the acetone extract was measured against a solvent blank (70% acetone) in a UV-ViS spectrophotometer at the wavelengths: 470 nm, 646 nm and 663 nm. The contents of photosynthetic pigments were calculated according to Lichtenthaler and Buschmann [126]. The results are expressed as mg/g DW.

Chl a, chl b, antheraxanthin, lutein, neoxanthin, violaxanthin and zeaxanthin were determined by the HPLC-PDA method according to Tausz et al. [127]. The HPLC analyses were performed using an HPLC-PDA chromatography system (HPLC Waters Alliance 2695 System, coupled with a 2996 photodiode array detector). Phenomenex SphereClone 5 µm 250 × 4.6 mm column was used. The run time was 35 min with a flow rate of 1 mL/min. Pigments were detected at a wavelength of 440 nm. Mobile phase A contained acetonitrile, doubly distilled water and methanol (100:10:5; *v*/*v*/*v*), while mobile phase B contained acetone and ethyl acetate (2:1; *v*/*v*). The gradient was from 10% (B) to 80% (B) in 17 min, followed by a hold for 5 min and a return to initial conditions after 5 min. Antheraxanthin, lutein, neoxanthin, zeaxanthin and violaxanthin were quantified in the equivalent of the related compound xanthophyll, because their standards were not available. The range of the xanthophyll standard curve was between 1 and 3 mg/L. The results are expressed as µg/g DW. Each sample vial was analyzed twice.

### 4.10. Determination of the Maximum Quantum Efficiency of PSII

The Fv/Fm was measured according to Rico-Cambron et al. [128]. Fv/Fm measurements were obtained using a handheld fluorometer PAR FluorPen FP 110/P (Photon Systems Instruments, Brno, Czech Republic) according to the settings recommended by the manufacturer: λ = 455 nm; flash-pulse: 900 μmol m^− 2^ s^− 1^, 30 μs; and super-pulse: 2400 μmol m^− 2^ s^− 1^. All measurements were performed between 11:00 and 14:00 on the 21st day of the experiment and conducted on fully developed sun-exposed fifth to seventh leaves below the apex. Measurements were performed on all plants included in the experiment and conducted in duplicate.

### 4.11. Statistics

The plant shoot-growth rates, which were calculated from three shoots from each plant, are presented by mean values (*n* = 30) and standard deviations (±SD) and were evaluated statistically by one-way analysis of variance (ANOVA) using the SPSS 21 software (SPSS Inc., Chicago, IL, USA). Significant differences between the mean values were determined using the Duncan post hoc test. Significant differences (*p* < 0.05) between the mean values were indicated by different letters. The Kolmogorov–Smirnov test was used to examine the normal distribution of data. The results of the biochemical analyzes (Cu content, total ASC content, DHA ratio, cysteine content, cystine ratio, total GSH content, GSSG ratio, TPC, photosynthetic and xanthophyll pigment content) are presented as mean values (±SD) of the analyses of plant material cultivated in two replicates. The measurements were performed at least twice for each sample and in duplicate. The Kolmogorov–Smirnov test was used to examine the normal distribution of data. One-way analysis of variance (ANOVA) was used to test for the differences between the effect of different cultivation time and variety on biochemical traits (SPSS Inc., Chicago, IL, USA). The post hoc Duncan test was used for the biochemical traits depending on the variety and sample term that were evaluated as significant (*p* < 0.05). Fv/Fm is presented as mean value (±SD). Fv/Fm values were measured in duplicate on each plant (*n* = 30). The post hoc Duncan test was used for the Fv/Fm depending on the variety and treatment with CuSO_4_ that were evaluated as significant (*p* < 0.05). Two-way analysis of variance (ANOVA) was conducted to identify the cultivation time dependency, the effect of CuSO_4_ treatment and variety on the biochemical traits of mulberry plants (SPSS Inc., Chicago, IL, USA). The Pearson correlation coefficient was also calculated between the observed biochemical parameters (Past4). A Ward diagram was constructed based on biochemical measurements of all sample terms, relative shoot-growth rate and Fv/Fm (Past4).

## 5. Conclusions

In the present study, the responses of three *M. alba* varieties (‘Budapest’, ‘Florio’, and ‘Giazzola’) and two *M. indica* varieties (‘Kokusou’ and ‘Shin-Tso’) to Cu-induced stress were evaluated. The results demonstrate clear species- and variety-specific differences in physiological, biochemical and photosynthetic responses to CuSO_4_ treatment. Overall, the tested *M. alba* varieties showed a greater capacity to maintain growth, redox homeostasis and photosynthetic performance under Cu exposure than the tested *M. indica* varieties. The integrated responses of growth, Cu accumulation, antioxidant metabolism, phenolic compounds, photosynthetic pigments and Fv/Fm indicate that ‘Florio’ and ‘Giazzola’ had the greatest potential capacity to tolerate the applied Cu stress. Their responses were characterized by more effective maintenance of ASC and GSH redox balance, sustained phenolic and carotenoid responses, and better preservation of PSII function. ‘Budapest’ showed an intermediate response, with activation of antioxidant and photoprotective mechanisms but less sustained maintenance during prolonged Cu exposure. In contrast, ‘Kokusou’ and ‘Shin-Tso’ showed stronger chlorosis and growth inhibition, progressive oxidation of ASC and thiol pools, greater pigment losses and a stronger decline in Fv/Fm, indicating greater sensitivity to Cu-induced stress. The coordinated changes observed across the measured parameters and the clustering analysis further suggest that Cu tolerance in mulberry is associated with effective redox regulation and photoprotection. However, because only one Cu concentration was tested and Cu content was not determined in roots, the results should be considered as evidence of potential relative tolerance. Given that certain *M. alba* varieties exhibit good potential tolerance to Cu, it would be valuable to evaluate Cu-induced stress tolerance of other closely related varieties. Moreover, future experiments should assess the phytoremediation potential of tolerant mulberry varieties in comparison with plant species already recognized as effective phytoremediators. Future studies using several Cu concentrations, root and shoot Cu determination, and analyses of Cu transporters, metallothioneins, phytochelatins and key antioxidant enzymes would help to clarify the mechanisms underlying the observed species- and variety-specific differences.

## Figures and Tables

**Table 1:** Mean content of chl a, chl b and total carotenoids (mg/g DW; ±SD) in leaves of *M. alba* and *M. indica* varieties at the 0th, 3rd, 7th and 21st days of the experiment (*n* = 120). Different lowercase letters (a–d) indicate significant differences (*p* < 0.05), determined by the post hoc Duncan test for comparing chl a, chl b and total carotenoid content in leaves of the same variety sampled at different sampling dates. Different uppercase letters (A–H) indicate significant differences (*p* < 0.05), determined by the post hoc Duncan test for comparing chl a, chl b and total carotenoid content in leaves of different varieties sampled at the same sampling date.

Genotype	Treatment	0 Days			3 Days			7 Days			21 Days		
		**chl a (mg/g DW)**											
‘Budapest’	control	4.051	±	0.023 Aa	4.020	±	0.017 ABa	4.059	±	0.035 Aa	4.048	±	0.025 Aa
‘Budapest’	CuSO_4_	4.051	±	0.023 Aa	3.413	±	0.077 Eb	2.847	±	0.027 Fc	2.063	±	0.045 Fd
‘Florio’	control	3.796	±	0.068 Ca	3.789	±	0.047 Ca	3.795	±	0.062 Ca	3.788	±	0.058 Ca
‘Florio’	CuSO_4_	3.796	±	0.068 Ca	3.543	±	0.011 Db	3.265	±	0.034 Dc	2.901	±	0.017 Dd
‘Giazzola’	control	3.889	±	0.019 BCa	3.944	±	0.012 ABa	3.924	±	0.014 Ba	3.905	±	0.018 Ba
‘Giazzola’	CuSO_4_	3.889	±	0.019 BCa	3.566	±	0.020 Db	3.076	±	0.064 Ec	2.646	±	0.018 Ed
‘Kokusou’	control	3.985	±	0.048 ABa	4.051	±	0.017 Aa	3.968	±	0.047 ABa	4.006	±	0.044 Aa
‘Kokusou’	CuSO_4_	3.985	±	0.048 ABa	2.719	±	0.025 Gb	1.509	±	0.013 Gc	0.701	±	0.007 Hd
‘Shin-Tso’	control	4.007	±	0.030 ABa	3.936	±	0.046 Ba	4.003	±	0.036 ABa	4.000	±	0.030 Aa
‘Shin-Tso’	CuSO_4_	4.007	±	0.030 ABa	2.881	±	0.019 Fb	1.568	±	0.003 Gb	0.819	±	0.031 Gc
		**chl b (mg/g DW)**											
‘Budapest’	control	1.519	±	0.021 ABa	1.518	±	0.028 BCa	1.510	±	0.026 Aa	1.514	±	0.027 ABa
‘Budapest’	CuSO_4_	1.519	±	0.021 ABa	1.380	±	0.027 Eb	1.243	±	0.013 Cc	0.951	±	0.008 Ed
‘Florio’	control	1.543	±	0.021 ABa	1.543	±	0.011 ABa	1.542	±	0.021 Aa	1.551	±	0.019 Aa
‘Florio’	CuSO_4_	1.543	±	0.021 ABa	1.482	±	0.008 CDb	1.406	±	0.012 Bc	1.278	±	0.006 Cd
‘Giazzola’	control	1.521	±	0.017 ABa	1.530	±	0.017 A–Ca	1.527	±	0.017 Aa	1.536	±	0.002 ABa
‘Giazzola’	CuSO_4_	1.521	±	0.017 ABa	1.434	±	0.013 Db	1.269	±	0.036 Cc	1.158	±	0.007 Dd
‘Kokusou’	control	1.573	±	0.010 Aa	1.573	±	0.010 Aa	1.544	±	0.021 Aa	1.551	±	0.022 Aa
‘Kokusou’	CuSO_4_	1.573	±	0.010 Aa	1.112	±	0.022 Fb	0.656	±	0.006 Dc	0.361	±	0.007 Fd
‘Shin-Tso’	control	1.497	±	0.016 Ba	1.502	±	0.012 BCa	1.515	±	0.017 Aa	1.505	±	0.013 Ba
‘Shin-Tso’	CuSO_4_	1.497	±	0.016 Ba	1.134	±	0.008 Fb	0.667	±	0.004 Dc	0.378	±	0.005 Fd
		**Total carotenoids (mg/g DW)**											
‘Budapest’	control	0.902	±	0.026 Aab	0.910	±	0.026 BCab	0.902	±	0.022 Bab	0.902	±	0.024 Cab
‘Budapest’	CuSO_4_	0.902	±	0.026 Aab	0.967	±	0.036 Ba	0.918	±	0.044 Bb	0.821	±	0.014 Dc
‘Florio’	control	0.913	±	0.019 Ac	0.904	±	0.021 BCc	0.906	±	0.014 Bc	0.906	±	0.021 Cc
‘Florio’	CuSO_4_	0.913	±	0.019 Ac	1.113	±	0.017 Ab	1.190	±	0.028 Aa	1.101	±	0.048 Ab
‘Giazzola’	control	0.911	±	0.010 Ad	0.913	±	0.010 BCd	0.912	±	0.008 Bd	0.914	±	0.004 Cd
‘Giazzola’	CuSO_4_	0.911	±	0.010 Ad	1.061	±	0.042 Ab	1.169	±	0.027 Aa	1.017	±	0.018 Bc
‘Kokusou’	control	0.880	±	0.009 Aa	0.882	±	0.012 Ca	0.888	±	0.012 Ba	0.886	±	0.013 Ca
‘Kokusou’	CuSO_4_	0.880	±	0.009 Aa	0.775	±	0.017 Db	0.636	±	0.018 Cc	0.444	±	0.015 Ed
‘Shin-Tso’	control	0.928	±	0.012 Aa	0.924	±	0.012 BCa	0.921	±	0.007 Ba	0.926	±	0.006 Ca
‘Shin-Tso’	CuSO_4_	0.928	±	0.012 Aa	0.853	±	0.011 Cb	0.654	±	0.015 Cc	0.459	±	0.014 Ed

**Table 2:** Mean content of neoxanthin, lutein, violaxanthin and antheraxanthin + zeaxanthin (µg/g DW; ±SD) in leaves of the varieties of *M. alba* and *M. indica* included in the experiment at the 0th, 3rd, 7th and 21st days of the experiment (*n* = 120). Different lowercase letters (a–d) indicate significant differences (*p* < 0.05), determined by the post hoc Duncan test for comparing neoxanthin, lutein, violaxanthin and A+Z content in leaves of plants of the same variety sampled at different sampling dates. Different uppercase letters (A–G) indicate significant differences (*p* < 0.05), determined by the post hoc Duncan test for comparing neoxanthin, lutein, violaxanthin and antheraxanthin + zeaxanthin content in leaves of mulberry plants of different varieties sampled at the same sampling date.

Genotype	Treatment	0 Day			3 Day			7 Day			21 Day		
		**Neoxanthin (µg/g DW)**											
‘Budapest’	control	45.9	±	0.8 ABa	46.0	±	1.0 BCa	46.0	±	0.7 Ca	46.0	±	1.0 BCa
‘Budapest’	CuSO_4_	45.9	±	0.8 ABa	42.9	±	1.0 Db	41.6	±	0.3 Ebc	39.4	±	0.8 Dc
‘Florio’	control	47.2	±	0.2 Ab	47.2	±	0.1 BCb	47.1	±	0.3 Cb	47.4	±	0.3 ABb
‘Florio’	CuSO_4_	47.2	±	0.2 Ab	48.2	±	1.3 ABb	52.0	±	0.5 Aa	48.7	±	0.2 Ab
‘Giazzola’	control	45.7	±	0.6 ABc	45.6	±	0.5 B-Dc	45.8	±	0.6 CDc	45.4	±	0.5 BCc
‘Giazzola’	CuSO_4_	45.7	±	0.6 ABc	50.3	±	0.9 Aa	49.8	±	0.2 Bab	47.4	±	1.7 ABb
‘Kokusou’	control	44.5	±	0.5 Ba	44.6	±	0.3 CDa	44.5	±	0.4 Da	44.5	±	0.4 Ca
‘Kokusou’	CuSO_4_	44.5	±	0.5 Ba	36.9	±	0.6 Fb	28.8	±	0.4 Gc	21.2	±	0.5 Ed
‘Shin-Tso’	control	46.3	±	0.3 Aa	46.5	±	0.2 BCa	46.5	±	0.3 Ca	46.8	±	0.3 A-Ca
‘Shin-Tso’	CuSO_4_	46.3	±	0.3 Aa	39.7	±	1.8 Eb	30.3	±	0.6 Fc	21.8	±	0.5 Ed
		**Lutein (µg/g DW)**											
‘Budapest’	control	367.0	±	3.2 Aa	364.3	±	3.6 Ba	365.6	±	4.9 BCa	364.3	±	2.9 Ba
‘Budapest’	CuSO_4_	367.0	±	3.2 Aa	367.9	±	11.9 Ba	383.6	±	6.7 Ba	365.5	±	6.0 Ba
‘Florio’	control	360.5	±	3.9 ABc	359.3	±	4.7 Bc	360.3	±	4.1 BCc	360.5	±	3.9 Bc
‘Florio’	CuSO_4_	360.5	±	3.9 ABc	414.8	±	11.5 Ab	452.8	±	13.4 Aa	421.2	±	12.5 Ab
‘Giazzola’	control	348.2	±	0.7 Bc	348.6	±	3.7 BCc	348.3	±	2.8 Cc	348.7	±	2.5 Bc
‘Giazzola’	CuSO_4_	348.2	±	0.7 Bc	424.0	±	13.1 Ab	455.6	±	14.4 Aa	408.9	±	0.9 Ab
‘Kokusou’	control	361.9	±	4.3 ABa	360.8	±	2.3 Ba	363.9	±	3.5 BCa	364.4	±	3.6 Ba
‘Kokusou’	CuSO_4_	361.9	±	4.3 ABa	329.3	±	6.9 Cb	268.5	±	2.4 Dc	228.6	±	3.7 Cd
‘Shin-Tso’	control	358.9	±	7.7 ABa	357.5	±	7.8 Ba	360.4	±	8.0 BCa	359.2	±	8.2 Ba
‘Shin-Tso’	CuSO_4_	358.9	±	7.7 ABa	331.9	±	7.2 Cb	276.9	±	9.4 Dc	230.3	±	3.4 Cd
		**Violaxanthin (µg/g DW)**											
‘Budapest’	control	38.4	±	0.2 Aa	38.5	±	0.3 Aa	38.6	±	0.2 BCa	38.4	±	0.3 Aa
‘Budapest’	CuSO_4_	38.4	±	0.2 Aa	34.6	±	0.5 Bb	31.8	±	0.9 Dc	29.4	±	1.1 Bd
‘Florio’	control	38.0	±	0.4 Ab	37.7	±	0.4 Ab	38.0	±	0.4 Cb	37.8	±	0.3 Ab
‘Florio’	CuSO_4_	38.0	±	0.4 Ab	37.3	±	0.8 Ab	43.1	±	0.8 Aa	38.8	±	1.0 Ab
‘Giazzola’	control	39.1	±	0.8 Aa	38.7	±	0.8 Aa	39.1	±	0.9 BCa	39.0	±	0.9 Aa
‘Giazzola’	CuSO_4_	39.1	±	0.8 Aa	39.7	±	1.7 Aa	40.6	±	1.4 Ba	39.6	±	0.9 Aa
‘Kokusou’	control	39.3	±	0.6 Aa	38.9	±	0.5 Aa	39.2	±	0.7 BCa	39.0	±	0.4 Aa
‘Kokusou’	CuSO_4_	39.3	±	0.6 Aa	28.2	±	0.5 Db	21.6	±	0.7 Ec	15.6	±	0.5 Cd
‘Shin-Tso’	control	39.0	±	0.3 Aa	39.0	±	0.5 Aa	39.1	±	0.2 BCa	38.9	±	0.3 Aa
‘Shin-Tso’	CuSO_4_	39.0	±	0.3 Aa	31.1	±	0.4 Cb	20.6	±	0.5 Ec	16.2	±	0.5 Cd
		**Antheraxanthin + zeaxanthin (µg/g DW)**											
‘Budapest’	control	3.4	±	0.1 Ad	3.4	±	0.1 Ed	3.4	±	0.1 Dd	3.4	±	0.1 Dd
‘Budapest’	CuSO_4_	3.4	±	0.1 Ad	8.3	±	0.2 Ca	6.3	±	0.2 Bb	4.6	±	0.1 Bc
‘Florio’	control	3.4	±	0.1 Ad	3.4	±	0.1 Ed	3.4	±	0.1 Dd	3.4	±	0.1 Dd
‘Florio’	CuSO_4_	3.4	±	0.1 Ad	11.0	±	0.1 Ba	9.1	±	0.1 Ab	5.8	±	0.1 Ac
‘Giazzola’	control	3.4	±	0.1 Ad	3.4	±	0.1 Ed	3.4	±	0.1 Dd	3.4	±	0.1 Dd
‘Giazzola’	CuSO_4_	3.4	±	0.1 Ad	11.7	±	0.2 Aa	9.1	±	0.3 Ab	6.0	±	2.1 Ac
‘Kokusou’	control	3.4	±	0.1 Ad	3.4	±	0.1 Ed	3.4	±	0.1 Dd	3.4	±	0.1 Dd
‘Kokusou’	CuSO_4_	3.4	±	0.1 Ad	6.1	±	0.1 Da	4.7	±	0.2 Cb	4.0	±	0.1 Cc
‘Shin-Tso’	control	3.4	±	0.1 Ad	3.4	±	0.0 Ed	3.4	±	0.1 Dd	3.4	±	0.1 Dd
‘Shin-Tso’	CuSO_4_	3.4	±	0.1 Ad	6.0	±	0.2 Da	4.7	±	0.1 Cb	4.1	±	0.1 Cc

**Table 3:** List of mulberry varieties included in the experiment and data on their origin and propagation for planting in the mulberry collection plantation, and propagation for the purpose of the experiment.

Genotype Name	Species	Origin	Obtained	Propagation for Planting in a Mulberry Collection Plantation	Propagation of Material from a Collection Plantation for Experimental Purposes
‘Budapest’	*Morus alba* (L.)	Hungary	Local variety, Budapest University of Veterinary Medicine campus (from the natural environment), Hungary	March 2015	June 2021
‘Florio’	*Morus alba* (L.)	Italy	Mulberry gene bank CREA Padua, Italy	December 2014	June 2021
‘Giazzola’	*Morus alba* (L.)	Italy	Mulberry gene bank CREA Padua, Italy	March 2015	June 2021
‘Kokusou’	*Morus indica* (L.)	Japan	Mulberry gene bank CREA Padua, Italy	March 2015	June 2021
‘Shin-Tso’	*Morus indica* (L.)	Japan	Hubmann, Austria	March 2014	June 2021

## Data Availability

The data used to support the findings of this study can be made available by the corresponding author upon request.

## Supplementary Materials

The following supporting information can be downloaded at https://www.mdpi.com/article/10.3390/plants15193070/s1, Table S1: Mean DHA ratio (% ±SD), mean cystine ratio (%; ±SD) and mean GSSG ratio (%; ±SD) in leaves of the varieties of *M. alba* and *M. indica* included in the experiment at the 0th, 3rd, 7th and 21st days of the experiment (*n* = 120). Different lowercase letters (a–d) indicate significant differences (*p* < 0.05), determined by the post hoc Duncan test for comparing DHA ratio, cystine ratio and GSSG ratio in leaves of plants of the same variety sampled at different sampling dates. Different uppercase letters (A–E) indicate significant differences (*p* < 0.05), determined by the post hoc Duncan test for comparing DHA ratio, cystine ratio and GSSG ratio in leaves of mulberry plants of different varieties sampled at the same sampling date. Table S2: Two-way ANOVA results on the effects of CuSO_4_ treatment, cultivation time and variety on observed biochemical characteristics.
