# Supplementary material for: Variety-Specific Responses of Mulberry to Copper-Induced Stress
Source: Plants (Basel). 2026 Oct 8;15(19):3070. doi: 10.3390/plants15193070 (PMC13667201; doi:10.3390/plants15193070)
Supplement: Supplementary file 1 [file plants-15-03070-s001.zip › plants-4564510-supplementary.pdf]

Table S1. Mean DHA ratio (% ±SD), mean cystine ratio (%; ±SD) and mean GSSG ratio (%; ±SD) in leaves of the varieties of *M. alba* and *M. indica* included in the experiment at 0th, 3rd, 7th and 21st day of the experiment (n = 120). Different lowercase letters (a–d) indicate significant differences ($p < 0.05$), determined by the post hoc Duncan test for comparing DHA ratio, cystine ratio and GSSG ratio in leaves of plants of the same variety sampled at different sampling dates. Different uppercase letters (A–E) indicate significant differences ($p < 0.05$), determined by the post hoc Duncan test for comparing DHA ratio, cystine ratio and GSSG ratio in leaves of mulberry plants of different varieties sampled at the same sampling date.

**genotype**	**treatment**	**DHA (%)**			
		**0 day**	**3 day**	**7 day**	**21 day**
**'Budapest'**	**control**	19.10 ± 0.37Ad	18.72 ± 0.16Ed	19.04 ± 0.51Ed	19.09 ± 0.41Ed
**'Budapest'**	**$CuSO_4$**	19.10 ± 0.37Ad	42.71 ± 0.45Bc	50.93 ± 0.50Ca	45.63 ± 0.69Cb
**'Florio'**	**control**	19.01 ± 0.69Ad	18.92 ± 0.27Ed	19.33 ± 0.56Ed	19.19 ± 0.17Ed
**'Florio'**	**$CuSO_4$**	19.01 ± 0.69Ad	34.82 ± 0.66Ca	26.15 ± 0.62Db	23.04 ± 0.44Dc
**'Giazzola'**	**control**	18.94 ± 0.39Ad	19.30 ± 0.12Ed	19.38 ± 0.27Ed	19.23 ± 0.36Ed
**'Giazzola'**	**$CuSO_4$**	18.94 ± 0.39Ad	32.09 ± 0.11Da	24.87 ± 0.45Db	22.28 ± 0.66Dc
**'Kokosou'**	**control**	19.19 ± 0.57Ad	18.95 ± 0.43Ed	19.41 ± 0.55Ed	19.37 ± 0.66Ed
**'Kokosou'**	**$CuSO_4$**	19.19 ± 0.57Ad	48.72 ± 0.85Ac	57.89 ± 1.35Ab	68.49 ± 1.69Aa
**'Shin-Tso'**	**control**	18.78 ± 0.50Ad	19.13 ± 0.30Ed	19.18 ± 0.18Ed	19.39 ± 0.31Ed
**'Shin-Tso'**	**$CuSO_4$**	18.78 ± 0.50Ad	48.96 ± 0.33Ac	55.23 ± 0.64Bb	65.90 ± 0.89Ba
		**cystine (%)**			
**'Budapest'**	**control**	10.01 ± 0.62Bd	13.57 ± 3.76Cd	11.62 ± 4.75Dd	13.76 ± 1.42Ed
**'Budapest'**	**$CuSO_4$**	10.01 ± 0.62Bd	39.33 ± 1.10ABc	56.42 ± 0.70Ba	48.80 ± 2.25Cb
**'Florio'**	**control**	18.43 ± 3.07Acd	14.48 ± 1.86Cd	12.21 ± 2.45Dd	13.08 ± 0.73Ed
**'Florio'**	**$CuSO_4$**	18.43 ± 3.07Acd	37.66 ± 1.44ABa	28.91 ± 1.24Cb	24.14 ± 3.25Dbc
**'Giazzola'**	**control**	12.57 ± 2.78ABd	13.33 ± 2.00Cd	17.83 ± 0.54Dcd	16.07 ± 2.15Ecd
**'Giazzola'**	**$CuSO_4$**	12.57 ± 2.78ABd	31.86 ± 2.90Ba	26.78 ± 1.03Cab	23.13 ± 2.68Dbc
**'Kokosou'**	**control**	17.98 ± 2.09Ad	16.11 ± 3.75Cd	16.13 ± 3.18Dd	16.09 ± 1.64Ed
**'Kokosou'**	**$CuSO_4$**	17.98 ± 2.09Ad	44.21 ± 1.37Ac	64.47 ± 1.34Ab	75.66 ± 1.84Aa
**'Shin-Tso'**	**control**	11.85 ± 0.27ABd	16.76 ± 3.42Cd	15.86 ± 2.21Dd	12.69 ± 0.60Ed
**'Shin-Tso'**	**$CuSO_4$**	11.85 ± 0.27ABd	41.46 ± 1.18Ac	57.41 ± 4.19ABb	68.44 ± 1.22Ba
		**GSSG (%)**			
**'Budapest'**	**control**	10.98 ± 0.21Bd	15.41 ± 2.12Dd	13.42 ± 3.14Cd	15.32 ± 2.59Ed
**'Budapest'**	**$CuSO_4$**	10.98 ± 0.21Bd	37.96 ± 1.18Bc	55.69 ± 1.48Aa	48.83 ± 0.58Cb
**'Florio'**	**control**	16.07 ± 1.85Ad	14.32 ± 1.21Dd	12.14 ± 1.40Cd	12.04 ± 0.74Ed
**'Florio'**	**$CuSO_4$**	16.07 ± 1.85Ad	33.70 ± 0.13BCa	28.44 ± 1.51Bb	24.15 ± 1.07Dc
**'Giazzola'**	**control**	12.86 ± 2.08ABd	14.82 ± 2.66Dd	16.82 ± 1.60Ccd	14.80 ± 2.31Ed
**'Giazzola'**	**$CuSO_4$**	12.86 ± 2.08ABd	31.34 ± 0.53Ca	26.21 ± 1.15Bab	21.04 ± 0.70Dbc
**'Kokosou'**	**control**	14.03 ± 1.76ABd	14.96 ± 1.40Dd	15.36 ± 2.17Cd	15.35 ± 1.82Ed
**'Kokosou'**	**$CuSO_4$**	14.03 ± 1.76ABd	43.50 ± 2.54Ac	61.49 ± 0.55Ab	76.68 ± 1.65Aa
**'Shin-Tso'**	**control**	15.56 ± 0.52ABd	17.65 ± 1.56Dd	14.67 ± 0.67Cd	15.15 ± 1.13Ed
**'Shin-Tso'**	**$CuSO_4$**	15.56 ± 0.52ABd	43.10 ± 0.43Ac	56.30 ± 3.22Ab	67.53 ± 1.48Ba

Table S2. Two-way ANOVA results on the effects of $CuSO_4$-treatment, cultivation time and variety on observed biochemical characteristics.

	**Sum of squares**	**Df**	**F**	**Sig**
**Cu**				
variety	77.030	4	439.333	**<0.001**
$CuSO_4$	29.304	1	668.534	**<0.001**
time	11.831	3	89.969	**<0.001**
variety * $CuSO_4$	11.365	4	64.817	**<0.001**
variety * time	4.670	12	8.879	**<0.001**
$CuSO_4$ * time	12.299	3	93.528	**<0.001**
variety * $CuSO_4$ * time	5.261	12	10.001	**<0.001**
**total ASC**				
variety	775243648	4	3139.591	**<0.001**
$CuSO_4$	3850.260	1	0.625	0.432
time	2603738.289	3	14.060	**<0.001**
variety * $CuSO_4$	76706882.6	4	310.648	**<0.001**
variety * time	42327266.8	12	57.139	**<0.001**
$CuSO_4$ * time	2555663.714	3	13.800	**<0.001**
variety * $CuSO_4$ * time	43099321.3	12	58.181	**<0.001**
**DHA**				
variety	3157.247	4	754.494	**<0.001**
$CuSO_4$	9724.681	1	9295.714	**<0.001**
time	3390.278	3	1080.243	**<0.001**
variety * $CuSO_4$	3160.809	4	755.346	**<0.001**
variety * time	1763.510	12	140.477	**<0.001**
$CuSO_4$ * time	3284.639	3	1046.583	**<0.001**
variety * $CuSO_4$ * time	1749.579	12	139.367	**<0.001**
**cysteine**				
variety	16995.555	4	3315.589	**<0.001**
$CuSO_4$	283.116	1	220.927	**<0.001**
time	402.220	3	104.623	**<0.001**
variety * $CuSO_4$	571.734	4	111.537	**<0.001**
variety * time	438.244	12	28.498	**<0.001**
$CuSO_4$ * time	348.999	3	90.780	**<0.001**
variety * $CuSO_4$ * time	413.849	12	26.912	**<0.001**
**cystine**				
variety	3386.626	4	55.447	**<0.001**
$CuSO_4$	15126.326	1	990.625	**<0.001**
time	5703.273	3	124.502	**<0.001**
variety * $CuSO_4$	2993.700	4	49.014	**<0.001**
variety * time	2617.827	12	14.287	**<0.001**
$CuSO_4$ * time	5441.588	3	118.790	**<0.001**
variety * $CuSO_4$ * time	2581.529	12	14.089	**<0.001**

**total GSH**				
variety	3988912.246	4	1163.674	**<0.001**
$CuSO_4$	791209.552	1	923.720	**<0.001**
time	901997.009	3	350.849	**<0.001**
variety * $CuSO_4$	1647880.031	4	480.731	**<0.001**
variety * time	817572.500	12	79.503	**<0.001**
$CuSO_4$ * time	994743.181	3	386.925	**<0.001**
variety * $CuSO_4$ * time	796306.195	12	77.435	**<0.001**
**GSSG**				
variety	3539.945	4	109.235	**<0.001**
$CuSO_4$	14110.430	1	1741.666	**<0.001**
time	5638.562	3	231.991	**<0.001**
variety * $CuSO_4$	3048.482	4	94.069	**<0.001**
variety * time	2559.896	12	26.331	**<0.001**
$CuSO_4$ * time	5182.794	3	213.239	**<0.001**
variety * $CuSO_4$ * time	2312.618	12	23.787	**<0.001**
**TPC**				
variety	201.456	4	311.351	**<0.001**
$CuSO_4$	678.919	1	4197.098	**<0.001**
time	317.409	3	654.078	**<0.001**
variety * $CuSO_4$	152.683	4	235.973	**<0.001**
variety * time	148.307	12	76.403	**<0.001**
$CuSO_4$ * time	299.541	3	617.256	**<0.001**
variety * $CuSO_4$ * time	141.062	12	72.671	**<0.001**
**chlorophyll a**				
variety	5.921	4	364.444	**<0.001**
$CuSO_4$	35.372	1	8708.927	**<0.001**
time	19.036	3	1562.239	**<0.001**
variety * $CuSO_4$	8.966	4	551.883	**<0.001**
variety * time	4.377	12	89.810	**<0.001**
$CuSO_4$ * time	19.115	3	1572.008	**<0.001**
variety * $CuSO_4$ * time	4.364	12	89.545	**<0.001**
**chlorophyll b**				
variety	1.406	4	399.373	**<0.001**
$CuSO_4$	3.728	1	4237.251	**<0.001**
time	2.128	3	806.125	**<0.001**
variety * $CuSO_4$	1.381	4	392.233	**<0.001**
variety * time	0.722	12	68.358	**<0.001**
$CuSO_4$ * time	2.112	3	799.926	**<0.001**
variety * $CuSO_4$ * time	0.671	12	63.595	**<0.001**
**chlorophyll a : chlorophyll b**				
variety	0.346	4	29.765	**<0.001**
$CuSO_4$	1.115	1	383.795	**<0.001**

time	0.697	3	79.929	**<0.001**
variety * $CuSO_4$	0.154	4	13.248	**<0.001**
variety * time	0.113	12	3.237	**<0.001**
$CuSO_4$ * time	0.722	3	82.748	**<0.001**
variety * $CuSO_4$ * time	0.153	12	4.388	**<0.001**
**total carotenoids**				
variety	0.809	4	156.901	**<0.001**
$CuSO_4$	0.013	1	10.189	**0.002**
time	0.147	3	37.960	**<0.001**
variety * $CuSO_4$	0.743	4	144.174	**<0.001**
variety * time	0.337	12	21.804	**<0.001**
$CuSO_4$ * time	0.147	3	38.079	**<0.001**
variety * $CuSO_4$ * time	0.347	12	22.462	**<0.001**
**neoxanthin**				
variety	0.002	4	275.798	**<0.001**
$CuSO_4$	0.001	1	432.064	**<0.001**
time	0.000	3	100.152	**<0.001**
variety * $CuSO_4$	0.001	4	209.100	**<0.001**
variety * time	0.001	12	34.921	**<0.001**
$CuSO_4$ * time	0.000	3	104.632	**<0.001**
variety * $CuSO_4$ * time	0.001	12	35.968	**<0.001**
**lutein**				
variety	0.070	4	126.156	**<0.001**
$CuSO_4$	$4.508*10^{-5}$	1	0.323	0.572
time	0.008	3	18.439	**<0.001**
variety * $CuSO_4$	0.086	4	153.987	**<0.001**
variety * time	0.038	12	22.713	**<0.001**
$CuSO_4$ * time	0.008	3	19.883	**<0.001**
variety * $CuSO_4$ * time	0.040	12	23.864	**<0.001**
**violaxanthin**				
variety	0.001	4	166.786	**<0.001**
$CuSO_4$	0.001	1	666.487	**<0.001**
time	0.000	3	112.151	**<0.001**
variety * $CuSO_4$	0.001	4	201.421	**<0.001**
variety * time	0.001	12	34.175	**<0.001**
$CuSO_4$ * time	0.000	3	109.247	**<0.001**
variety * $CuSO_4$ * time	0.001	12	33.707	**<0.001**
**antheraxanthin**				
variety	$7.421*10^{-8}$	4	136.586	**<0.001**
$CuSO_4$	$1.281*10^{-7}$	1	942.724	**<0.001**
time	$9.029*10^{-8}$	3	221.562	**<0.001**
variety * $CuSO_4$	$8.311*10^{-8}$	4	152.954	**<0.001**
variety * time	$3.759*10^{-8}$	12	23.060	**<0.001**

$CuSO_4$ * time	$9.029*10^{-8}$	3	221.562	**<0.001**
variety * $CuSO_4$ * time	$3.759*10^{-8}$	12	23.060	**<0.001**
**zeaxanthin**				
variety	$4.670*10^{-5}$	4	309.153	**<0.001**
$CuSO_4$	0.000	1	4801.977	**<0.001**
time	0.000	3	969.069	**<0.001**
variety * $CuSO_4$	$4.678*10^{-5}$	4	309.691	**<0.001**
variety * time	$2.550*10^{-5}$	12	56.271	**<0.001**
$CuSO_4$ * time	0.000	3	962.141	**<0.001**
variety * $CuSO_4$ * time	$2.526*10^{-5}$	12	55.753	**<0.001**
**antheraxanthin + zeaxanthin**				
variety	$5.044*10^{-5}$	4	324.701	**<0.001**
$CuSO_4$	0.000	1	4920.719	**<0.001**
time	0.000	3	996.931	**<0.001**
variety * $CuSO_4$	$5.080*10^{-5}$	4	327.038	**<0.001**
variety * time	$2.740*10^{-5}$	12	58.787	**<0.001**
$CuSO_4$ * time	0.000	3	989.994	**<0.001**
variety * $CuSO_4$ * time	$2.715*10^{-5}$	12	58.257	**<0.001**
**violaxanthin / antheraxanthin + zeaxanthin**				
variety	0.876	4	1.578	0.188
$CuSO_4$	732.023	1	5272.810	**<0.001**
time	257.236	3	617.629	**<0.001**
variety * $CuSO_4$	2.672	4	4.812	**0.002**
variety * time	16.872	12	10.128	**<0.001**
$CuSO_4$ * time	250.762	3	602.086	**<0.001**
variety * $CuSO_4$ * time	15.290	12	9.178	**<0.001**
